# Genomic Insights Into the Multimetal Resilience and Biofilm‐Templated Nanorod Biosynthesis of *Stenotrophomonas bentonitica* BII‐R7: Bioremediation and Green Nanotechnology Implications

**DOI:** 10.1111/1751-7915.70422

**Published:** 2026-09-06

**Authors:** Eduardo Perez‐Muelas, Miguel Angel Ruiz‐Fresneda, Guillermo Lazuen‐Lopez, Tatiana Lopez‐Perez, Fouad Eddaoudi‐Lakraichi, Mohammed Bakkali, Mohamed Larbi Merroun

**Affiliations:** ^1^ Department of Microbiology, Campus Fuentenueva University of Granada Granada Spain; ^2^ Department of Genetics, Campus Fuentenueva University of Granada Granada Spain

**Keywords:** biofilm, bioremediation, comparative genomics, se nanorods, *Stenotrophomonas*

## Abstract

While microbial metal reduction is widely documented, the genomic determinants that govern the morphological transition from disordered phases to structured nanocrystals remain elusive. Here, we present an integrative study of *Stenotrophomonas bentonitica* BII‐R7, a strain exhibiting exceptional metal resistance and the unique capacity to synthesize crystalline trigonal selenium (t‐Se) nanorods. Comparative pangenomic analysis of 38 *Stenotrophomonas* strains revealed that BII‐R7 possesses a notably large accessory genome of 2311 exclusive singletons. We identify a specialized genomic toolkit, absent in all related strains, comprising key metal resistance determinants (e.g., *copB, copF,* and *czcA*) alongside extracellular remodelling enzymes (Wzyligase and GH92‐glycosyl hydrolase). This unique repertoire confers BII‐R7 with significantly higher Cu and Ni tolerance compared to related *Stenotrophomonas* species, which we hypothesize is fundamental for maintaining metabolic activity in polymetallic environments. RT‐qPCR and functional assays confirm that these singletons are not only upregulated under metal stress (e.g., *czcA*: 42.2‐fold) but are also consistent with a critical role in maintaining biofilm resilience. Crucially, we propose a mechanistic model where this unique genetic repertoire governs the assembly of a compositionally distinctive Extracellular Polymeric Substance (EPS). Using a three‐state (biofilm, planktonic, EPS‐depleted) experiment, we provide direct phenotypic evidence that an intact EPS matrix is required for the efficient transition from amorphous nanospheres to highly ordered crystalline nanorods, and we propose that it acts as a molecular template directing the anisotropic growth of selenium. By bridging genomics and bionanotechnology, this work positions BII‐R7 as a promising candidate for sustainable green synthesis and bioremediation, while defining the targeted gene‐knockout and complementation experiments now required to establish direct causal roles for the candidate determinants.

## Graphical abstract

Biofilm extracellular polymeric substances (EPS) in *Stenotrophomonas bentonitica* BII‐R7 direct the formation of crystalline trigonal selenium nanorods. Comparative genomics identifies a unique genetic toolkit underpinning EPS composition and metal resilience, positioning this strain as a platform for bioremediation and green nanomaterial synthesis.

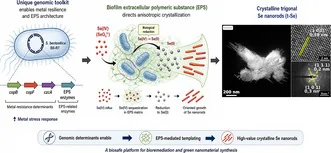

## 1 Introduction

The anthropogenic mobilization of selenium into aquatic and terrestrial ecosystems constitutes a growing global challenge. Industrial activities including coal combustion, mining operations, and oil refining, along with the application of seleniferous agricultural amendments release selenium predominantly as the water‐soluble oxyanions selenate [Se(VI)] and selenite [Se(IV)]. These Se species accumulate in groundwater and food webs at concentrations harmful to wildlife and humans (Fernández‐Martínez and Charlet 2009). Conventional physicochemical treatment strategies remain costly, energy‐intensive, and generate hazardous secondary waste. Dissimilatory microbial reduction of Se oxyanions to insoluble elemental selenium [Se(0)] offers a more sustainable alternative, and its integration into bioreactor‐based treatment systems is an active area of research (Nancharaiah and Lens 2015; Ruiz‐Fresneda, Fernández‐Cantos, et al. 2023).

The biotechnological value of biogenic Se(0) lies in its existence as selenium nanoparticles (SeNPs); however, this is not equivalent across its known structural forms. The majority of bacteria that reduce selenite produce amorphous red nanospheres (a‐Se), a metastable, disordered allotrope with limited conductivity, poor thermodynamic stability, and minimal industrial utility beyond bulk selenium immobilization (Ojeda et al. 2020). Crystalline trigonal selenium (t‐Se), by contrast, presents entirely different material properties: its one‐dimensional helical chain structure confers exceptional photoconductive and semiconducting properties, anisotropic optical characteristics, and thermodynamic stability that render it applicable in photovoltaic devices, photodetectors, rectifiers, and advanced nanofabrication (Ruiz‐Fresneda et al. 2018; Ibragimov et al. 2000). From a bioremediation standpoint, crystalline t‐Se nanorods also display superior settleability and filterability compared to amorphous nanospheres (Dessì et al. 2016).

Biological synthesis of crystalline t‐Se nanorods is, however, an exceptionally rare phenotype, documented in only a handful of bacterial species. In all reported cases the mechanistic basis for crystalline nanorod formation, as opposed to the amorphous nanosphere, remains poorly understood. A compelling but largely untested hypothesis is that the extracellular polymeric substance (EPS) matrix of bacterial biofilms plays an instructive role in this allotropic transition. EPS matrices are compositionally complex, containing polysaccharides, structural proteins, nucleic acids, and lipids, providing metal‐chelating functional groups and structurally organized scaffolds for biomineralization (Flemming and Wingender 2010; Decho 2010). In analogous inorganic systems, such as the transformation of amorphous calcium carbonate into crystalline calcite in tufa‐depositing biofilms, EPS‐mediated polymorph selection and crystal habit control are well documented (Broughton 2023). Whether a bacterial biofilm EPS matrix can analogously govern the selenium allotropic transition, and if so, through what molecular mechanisms has not been addressed. Previous work on biogenic Se(0) has characterized the reduction chemistry and, in a few organisms, the transcriptomic response to selenium, but has not experimentally separated the extracellular contribution from the intracellular enzymatic one, nor tested whether an intact EPS matrix is necessary for crystalline nanorod formation; as a result, the determinants of allotrope selection have remained largely inferential.

*Stenotrophomonas bentonitica* BII‐R7, isolated from bentonite clay formations in southeastern Spain, is currently the only documented member of the *Stenotrophomonas* genus and one of the very few bacteria globally capable of direct extracellular biosynthesis of crystalline t‐Se nanorods (Pinel‐Cabello et al. 2021; Ruiz‐Fresneda, Martinez‐Moreno, et al. 2023). The strain additionally exhibits an unusual breadth of multimetal tolerance, having been shown to immobilize uranium, europium, and curium under environmentally relevant conditions (Ruiz‐Fresneda et al. 2018, 2020). Classified as Biosafety Level 1, BII‐R7 lacks the virulence determinants characteristic of the opportunistic pathogen *S. maltophilia* , positioning it as a safe platform for environmental biotechnology applications (Sánchez‐Castro et al. 2017). Despite extensive phenotypic characterization, the genomic basis for BII‐R7's dual capacity, exceptional multimetal tolerance combined with EPS‐templated crystalline nanorod synthesis, has not been systematically investigated. We note at the outset that enhanced Cu and Ni resistance is not proposed to contribute chemically to crystallization; rather, it is proposed to be permissive, sustaining an intact, EPS‐producing biofilm under the polymetallic conditions that would otherwise suppress biofilm formation, thereby preserving the extracellular scaffold on which crystalline nanorod formation depends. Alternative or complementary contributions to allotrope selection, including intracellular redox control of nucleation, the activity of dedicated reductases, and slow thermodynamic ripening of metastable phases, are considered alongside the EPS hypothesis throughout this work.

To address these questions, we designed an integrated investigation combining: (i) a controlled three‐state physiological experiment to test directly the role of EPS in Se(0) allotrope determination; (ii) comparative pangenomic analysis of 38 *Stenotrophomonas* genomes to identify BII‐R7's exclusive genetic toolkit; (iii) quantitative biofilm assays under poly‐metal stress; (iv) functional metal tolerance and gene expression assays to validate genomic predictions; and (v) structural analysis of candidate exclusive EPS‐biosynthesis proteins. By connecting biofilm phenotype, genomic architecture, and nanomaterial output across multiple levels of resolution, this study clarifies the phenotypic basis of this dual capacity and proposes, rather than definitively establishes, a genomics‐informed mechanistic framework linking BII‐R7's unique genotype and its capacity for precision green synthesis of crystalline Se nanomaterials. We emphasize that the genomic components of this framework are correlative and hypothesis‐generating: they nominate the strongest candidate determinants and yield specific, testable predictions for the targeted mutagenesis required to establish causation.

## 2 Materials and Methods

### 2.1 Bacterial Strain and Culture Conditions

The focal strain of this study, *Stenotrophomonas bentonitica* BII‐R7, was originally isolated from bentonite clay from the Cortijo de Archidona deposit (Almería, Spain) and has been extensively characterized for multimetal resistance and nanomaterial biosynthesis (Sánchez‐Castro et al. 2017; Ruiz‐Fresneda et al. 2018; Ruiz‐Fresneda, Fernández‐Cantos, et al. 2023). Three additional environmentally derived strains were included for comparative biofilm and metal tolerance assays: *S. chelatiphaga* DSM 21508 (sewage sludge isolate), *S. pavanii* DSM 25135 (sugarcane stems), and *S. rhizophila* DSM 14405 (canola rhizosphere). *S. maltophilia* strains were excluded from all comparative assays due to their opportunistic pathogen status. Selection criteria for the four strains included: (i) environmental origin with no known human pathogenicity, (ii) representation of distinct ecological niches, (iii) phylogenetic proximity to BII‐R7 (Sánchez‐Castro et al. 2017), and (iv) documented metal tolerance or bioremediation capacity.

All strains were routinely maintained on Luria‐Bertani Broth (LB) agar plates at 28°C. Liquid cultures were grown in LB broth at 28°C with continuous shaking at 180 rpm. Working stocks were prepared from overnight cultures and standardized to an optical density at 600 nm (OD_600_) of 0.6 in fresh LB medium for all subsequent assays, unless otherwise stated.

### 2.2 Preparation of Cellular States and Selenium Exposure

To establish a causal link between EPS matrix composition and selenium nanorod morphogenesis, we designed a differential state experiment comparing three cellular conditions following the procedures of Priyadarshanee and Das (2024): (i) Biofilm‐associated cells: cultures incubated 48 h at 28°C in LB broth with 3 mm glass beads to promote surface‐attached biofilm. (ii) Planktonic cells: cultures grown under continuous shaking (180 rpm) for 24 h to prevent biofilm development. (iii) EPS‐depleted cells: biofilm‐associated cells treated with formaldehyde (0.4%, 1 h) followed by NaOH (0.5 M, 3 h) to remove extracellular polymers while preserving cell viability.

All three cellular states were subsequently exposed to 2 mM sodium selenite (Na_2_SeO_3_; analytical grade) in fresh LB broth (initial pH 7.0 ± 0.2) at 28°C under agitation (100 rpm) for 168 h. Selenite reduction was monitored visually by the appearance of characteristic red (amorphous Se(0)) or grey (trigonal Se(0)) precipitates. Samples were collected at 168 h for structural characterization.

### 2.3 Se Nanoparticle Structural Characterization

Crystalline phase identification and particle size estimation were performed using XRD analysis. After 168 h of exposure to 2 mM Se(IV), biogenic selenium nanoparticles together with the associated biomass were harvested by centrifugation (10,000 rpm, 10 min). Pellets were washed three times with sterile ultrapure water, with resuspension and re‐centrifugation between successive washes to remove residual medium and unreacted selenite, and were then air‐dried at 28°C for 168 h. Dried material was stored in sealed tubes at 4°C in the dark until analysis. Diffractograms were acquired using a Bruker D8 Advanced diffractometer equipped with a LINXEYE detector (Cu Kα radiation, *λ* = 1.5406 Å). Crystalline phases were identified by comparison with reference patterns from the Crystallography Open Database (COD). Average crystallite sizes were calculated using the Scherrer equation.

To characterize the molecular interactions between EPS components and selenium species, attenuated total reflectance Fourier‐transform infrared spectroscopy (ATR‐FTIR) was performed on washed pellets from biofilm‐associated cells exposed to 2 mM Se(IV) for 168 h, and on non‐exposed biofilm cultures as controls. Sample preparation followed the protocol described by Ruiz‐Fresneda et al. (2024). Spectra were recorded using a JASCO 6200 spectrometer (4000–400 cm^−1^, 4 cm^−1^ resolution, 50 scans/sample).

Morphology, elemental composition, and crystallographic characterization were performed using a Thermo Fisher Scientific TALOS F200X HAADF‐STEM microscope. Sample preparation followed Ruiz‐Fresneda, Fernández‐Cantos, et al. (2023). STEM specimen holders were plasma‐cleaned prior to analysis to minimize carbon contamination. Structural characterization employed selected‐area electron diffraction (SAED) for phase identification and high‐resolution TEM (HRTEM) combined with fast Fourier transform (FFT) analysis for lattice spacing determination and crystallographic orientation mapping.

### 2.4 Biofilm Assays

Four environmentally derived *Stenotrophomonas* strains (*S. bentonitica* BII‐R7, *S. rhizophila* DSM 14405, *S. chelatiphaga* DSM 21508, and *S. pavanii* DSM 25135) were selected for experimental validation. Genomes of *Stenotrophomonas* strains were screened for genes involved in quorum sensing (*rpfF*, *ax21*, *smoR*), polysaccharide production (*spgM*, *rmlA*, *rmlC*), and flagella assembly (*fleQ*, *flgE*/*G*/*K*/*I*, *flhA*, *fliF*/*I*/*K*/*M*/*N*/*O*/*A*, *fimV*), following Li et al. (2023). Gene presence/absence patterns were visualized to identify strain‐specific differences in biofilm regulatory architecture.

Biofilm formation capacity was quantified using the crystal violet staining method adapted from Li et al. (2023). Briefly, isolates were cultured in LB broth for 72 h at 28°C, which is optimal for the selected four strains. Aseptic LB broth served as the blank control. Each isolate was then diluted in fresh LB broth to achieve a cell density equivalent to 10^8^ CFU/mL. Subsequently, 100 μL of the diluted culture was transferred into each well of a 96‐well round‐bottom microtiter plate and incubated at 28°C for 72 h. After incubation, the culture supernatants were carefully discarded, and each well was washed three times with 200 μL of sterile saline solution (NaCl 0.9%), followed by drying at 28°C for 1 h.

To stain the samples, the crystal violet staining procedure was performed as described in Li et al. (2023). To assess metal‐responsive biofilm modulation, the assay was performed under five conditions: control (no metal addition) and 2 mM concentrations of copper (Cu(NO_3_)_2_), selenium (Na_2_SeO_3_), nickel (Ni(NO_3_)_2_), and cadmium (Cd(NO_3_)_2_). This concentration was selected based on BII‐R7's demonstrated tolerance threshold from growth curve analyses.

### 2.5 Genome Sequencing and DNA Extraction

The Gram‐negative *S. bentonitica* BII‐R7, previously sequenced and characterized by Sánchez‐Castro et al. (2017), was selected as the main focus of this comparative analysis. Since the draft genome available did not meet the established quality thresholds, the strain was re‐sequenced to improve data reliability. Genomic DNA was extracted following growth in Luria‐Bertani (LB) broth medium (tryptone 10 g l^−1^, yeast extract 5 g l^−1^, and NaCl 10 g l^−1^, pH 7.0 ± 0.2), as described by Martín‐Platero et al. (2007). Subsequently, a genomic library with an insert size of 350 bp was prepared and sequenced using the Illumina HiSeq 2000 platform. All of the pre‐annotation analyses were performed on the Galaxy Web server (https://usegalaxy.org), using default parameters. Quality control of the raw reads was performed by FastQC version 0.72 (Andrews 2010). Sequence reads were *de novo* assembled using Unicycler version 0.5.1.0 (Wick et al. 2017), and the assembly quality was assessed using QUAST version 0.4.6.3 (Mikheenko et al. 2018).

### 2.6 Genome Dataset and Pangenome Analysis

To identify genetic determinants unique to *S. bentonitica* BII‐R7, we constructed a phylogenetically and ecologically representative dataset of *Stenotrophomonas* genomes. A total of 38 genomes from the genus were retrieved from the National Center for Biotechnology Information (NCBI) database. Strain selection prioritized reference and functionally characterized genomes across ecological and phylogenetic breadth, including representatives from human clinical, rhizospheric, and soil environments. For example, *S. maltophilia* K279a and FDAARGOS isolates serve as clinical references; *S. rhizophila* DSM 14405 is a known plant growth‐promoting and biocontrol strain; *S. maltophilia* SeITE02 has demonstrated selenium tolerance and nanomaterial biosynthesis; and *S. bentonitica* BII‐R7 has been extensively characterized for multimetal resistance and previously subjected to transcriptomic, proteomic, and spectroscopic analysis. Genome inclusion required completeness > 97% and contamination < 2% as assessed by CheckM (Parks et al. 2015).

Pangenome analysis was performed using Roary version 3.12.0 (Page et al. 2015), processing GFF3 annotation files generated by Prokka (Seemann 2014), with BLASTp all‐against‐all comparisons at a 90% sequence identity threshold. Genes were categorized as core (present in all 38 strains), soft‐core (37 strains), shell (5–36 strains), or cloud (< 5 strains). To identify strain‐specific genes, the gene_presence_absence.csv output was analysed using the union and difference commands. Phylogenetic analysis was performed on core genome SNPs with recombination regions excluded; the remaining SNPs were concatenated and used to construct a maximum likelihood phylogenetic tree with FastTree version 2.1 (Price et al. 2010), visualized with iTOL v7 (Letunic and Bork 2024).

### 2.7 Detection of Genomic Islands and Potential Horizontal Gene Transfer (HGT) Events Using Sliding Windows

Candidate genomic islands were computationally identified using a sliding window approach (window size = 2000 bp, step size = 2000 bp) based on GC content deviation. Regions showing deviations greater than two standard deviations from the genome mean were flagged as potential genomic islands. To infer potential HGT origins, all predicted island sequences were subjected to BLASTN searches against the NCBI nucleotide (NT) database (release 2023); islands exhibiting high sequence similarity (> 80% identity, > 70% coverage) to taxa outside *Stenotrophomonas* were flagged as candidates for foreign origin. The 10 kb flanking regions of each island were screened for mobility‐associated genetic elements, including tRNA genes, integrases, transposases, recombinases, conjugation operons, phage‐related sequences, plasmid‐associated genes, mobilization proteins (e.g., mob genes), and CRISPR‐Cas components. Islands separated by less than 5000 bp were merged into single contiguous loci.

### 2.8 Identification and In Silico Structural Profiling of Exclusive EPS‐Biosynthesis Proteins

Genes in BII‐R7's exclusive gene set with predicted roles in heavy metal resistance were identified through BLASTX searches against the BacMet database v2.0 (Pal et al. 2014), retaining only hits meeting stringent criteria: *E*‐value < 0.01, sequence similarity > 75%, and alignment coverage > 80%. All BacMet‐identified genes were additionally cross‐referenced against NCBI RefSeq and UniProt (accessed October 2024) using BLASTp to ensure annotations reflected current nomenclature. Genes exclusive to BII‐R7 or present as divergent allelic variants were prioritized for functional characterization.

Transmembrane topology and signal peptide architecture were predicted using DeepTMHMM v1.0.25 (Hallgren et al. 2022; https://dtu.biolib.com/DeepTMHMM) with default parameters. Three‐dimensional structural models were generated using AlphaFold3 via the AlphaFold Server (Abramson et al. 2024; https://alphafoldserver.com), with default settings. Model confidence was assessed using per‐residue predicted local distance difference test (pLDDT) scores and pairwise predicted aligned error (PAE) matrices extracted from the server JSON outputs. Extracellular loop boundaries were defined from the DeepTMHMM GFF3 annotation; per‐loop mean pLDDT and PAE values were computed with custom Python scripts. Residues with pLDDT < 70 were classified as structurally uncertain. Amino acid composition of functionally relevant loop regions and metal‐coordinating residue content (Asp, Glu, His, Cys) we calculated directly from the protein sequence.

### 2.9 Metal Tolerance Assays

To validate genomic predictions of enhanced metal tolerance in BII‐R7, growth under copper and nickel stress was quantitatively assessed for the four *Stenotrophomonas* strains listed above. Nickel stress was imposed using nickel(II) nitrate hexahydrate (Ni(NO_3_)_2_·6H_2_O), and copper stress using copper(II) nitrate trihydrate (Cu(NO_3_)_2_·3H_2_O). Standardized cultures (OD_600_ = 0.6) were inoculated into 96‐well microplates containing LB broth supplemented with metal compounds at final concentrations of 0, 0.25, 0.5, 1, 2, 4, 6, and 8 mM. Each condition was tested in triplicate. Plates were incubated at 28°C with continuous shaking at 180 rpm, and bacterial growth was monitored by OD_600_ every 2 h over a 24‐h period. Growth curves were normalized to initial OD_600_ values.

### 2.10 Quantitative Real‐Time PCR (RT‐qPCR) Analysis

To establish transcriptional evidence linking BII‐R7‐exclusive genes to metal stress responses, RT‐qPCR was performed for three representative resistance determinants (*czcA*, *copB*, *copF*) following exposure to 2 mM nickel or copper for 16 h under aerobic conditions. Untreated cultures grown under identical conditions served as the control group. All experiments were conducted using four biological replicates (*n* = 4). Total RNA was extracted using the RNeasy Mini Kit (Qiagen), including on‐column DNase I digestion to eliminate genomic DNA contamination. RNA quantity and purity were assessed using NanoDrop spectrophotometry, and integrity was verified via agarose gel electrophoresis. Complementary DNA (cDNA) was synthesized from 1 μg of total RNA using the iScript cDNA Synthesis Kit (Bio‐Rad). RT‐qPCR was performed using SsoAdvanced Universal SYBR Green Supermix (Bio‐Rad) in a CFX96 Touch Real‐Time PCR Detection System (Bio‐Rad) following the manufacturer's protocol. Expression levels of the target genes (*czcA*, *copB*, and *copF*) were normalized against the *16S rRNA* gene, and relative expression was calculated using the 2^−ΔΔCT^ method. Statistical comparisons between metal‐treated and control groups were made using one‐way ANOVA followed by Tukey's post hoc test (*p* < 0.05). To complement these de novo expression data, previously published transcriptomic (Pinel‐Cabello et al. 2023) and proteomic (Pinel‐Cabello et al. 2021) datasets for BII‐R7 were retrieved and reanalyzed to validate expression patterns of additional pangenome‐predicted singletons under selenium stress.

## 3 Results and Discussion

### 3.1 Biofilm‐Associated EPS Templates in the Biosynthesis Selenium Nanostructures

The central objective of this study was to determine whether the extracellular polymeric substance (EPS) matrix of bacterial biofilms could direct the structural outcome of selenium biotransformation. Specifically, we investigated whether intact biofilms produce crystalline trigonal selenium (t‐Se) nanorods, while the same organism in a planktonic or EPS‐depleted state yields only amorphous selenium.

The macroscopic evaluation of the cultures revealed a distinct colour change from colourless to red or grey precipitates, indicating the formation of different selenium allotropes depending on the cellular state (Figure S1). Planktonic and EPS‐depleted cultures produced uniformly bright red precipitates, the characteristic and widely reported colour of amorphous Se(0) (a‐Se), in which selenium atoms are arranged in disordered polymeric ring and chain structures that absorb strongly in the blue‐green region of the visible spectrum (Lenz and Lens 2009). Biofilm‐associated cultures, by contrast, produced precipitates ranging in colour from dark red to metallic grey, a colour transition that is well established as an indicator of the transition from amorphous to the more ordered trigonal Se(0) allotrope (t‐Se), in which one‐dimensional helical selenium chains pack into a crystalline lattice with metallic lustre and reduced light scattering (Ruiz‐Fresneda et al. 2018; Ojeda et al. 2020). This macroscopic distinction between conditions is unambiguous and reproducible, immediately implicating the EPS matrix as the primary determinant of the allotropic outcome of Se(IV) biotransformation. The colour difference also has direct practical significance: the metallic‐grey appearance of the biofilm‐derived product signals improved settleability and recoverable crystalline quality, both of which are critical performance metrics in bioreactor‐based selenium remediation systems (Dessì et al. 2016). It is important to note that the three cellular states were prepared and incubated under identical physicochemical conditions so that the observed differences in Se(0) colour and, hence, allotropic form can be attributed exclusively to the cellular state and, within that, to the presence or absence of an intact EPS matrix. The EPS‐depleted condition controls for the cellular metabolic capacity for Se(IV) reduction in the absence of EPS structure, confirming that reduction per se does not require EPS but that EPS is required to direct the structural form of the Se reduction product.

#### 3.1.1 X‐Ray Diffraction: Confirmation of Trigonal Se Crystallinity Exclusively in the Biofilm Condition

**FIGURE 1:**
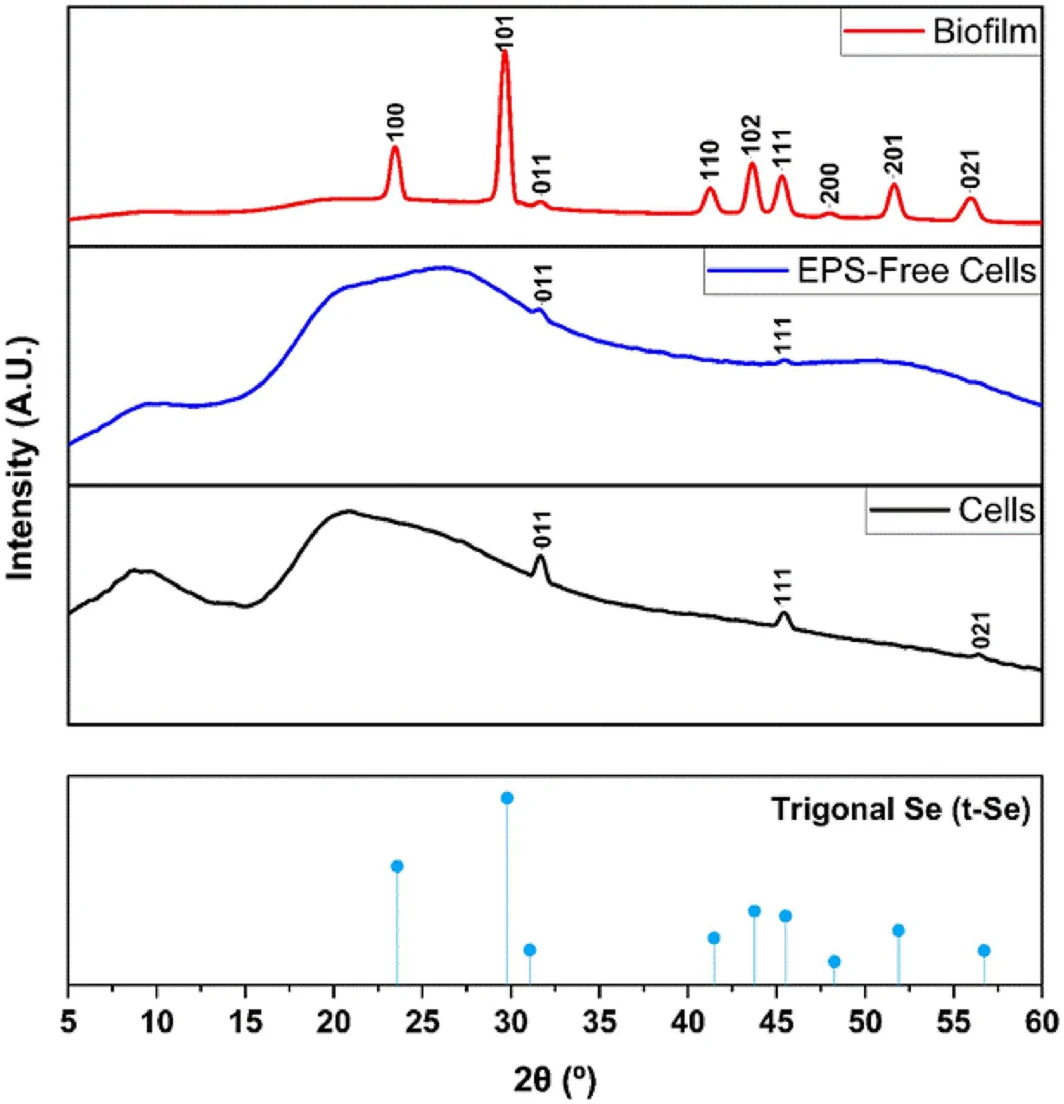
X‐ray diffraction (XRD) patterns of Se nanoparticles biosynthesized by *S. bentonitica* under three cellular conditions: (red) intact biofilms (with native EPS), (black) planktonic cells, and (blue) EPS‐depleted cells. X‐ray reflections of t‐Se (COD‐9008579) obtained from Crystallography Open Database (http://www.crystallography.net/cod/) are shown at the bottom.

X‐ray diffraction analysis provided unambiguous confirmation of the macroscopic colour observations and revealed a gradient of crystallinity across the three cellular states (Figure 1). The diffractogram obtained from the biofilm‐associated sample displayed multiple sharp, well‐resolved reflections at 2θ values of approximately 23.5°, 29.7°, and 43.6°, assigned respectively to the (100), (011), and (110) crystallographic planes of trigonal selenium. These assignments were made by comparison with the reference diffraction pattern for t‐Se retrieved from the Crystallography Open Database (COD ID: 9008579). Additional weaker reflections consistent with higher‐order t‐Se planes (102, 111, 200, 201, 021) are also visible in the biofilm pattern, further confirming phase purity and crystallographic completeness of the trigonal phase.

In contrast, the XRD pattern from Se‐treated planktonic cells showed weak but detectable t‐Se reflections at the same 2θ positions, superimposed on broad, asymmetric amorphous scattering peaks at approximately 31°, 43°, and 56°, indicating a mixed‐phase product dominated by amorphous Se(0). The EPS‐depleted condition showed an even more pronounced lack of crystallinity, with t‐Se peaks not detectable above baseline noise while amorphous scattering, the hallmark of Se(0) with short‐range but no long‐range periodic order, dominated the spectra. The systematic decrease in t‐Se peak intensity across the biofilm→planktonic→EPS‐depleted series, while Se reduction capacity is retained in all three states, indicates that an intact native EPS matrix is required to drive efficient conversion to the crystalline phase. The minor crystalline fraction retained in planktonic and EPS‐depleted conditions likely reflects residual surface‐associated EPS proteins or flagellar/pilus structures, rather than any EPS‐independent crystallization mechanism.

The XRD data indicate that an intact, native EPS matrix is required to direct the biotransformation product toward the predominant formation of the crystalline t‐Se allotrope. Crucially, this effect is not replicated to the same extent by cellular enzymatic activity alone or by residual surface‐associated biomolecules. The exclusive production of high‐intensity t‐Se reflections by the biofilm condition is noteworthy in the context of the broader bacterial SeNP literature. Although thermal annealing or prolonged aging of a‐Se can drive a slow, thermodynamically favourable transition toward t‐Se, this process typically requires temperatures above 100°C and hours to days at elevated temperatures (Ruiz‐Fresneda et al. 2018). The observation of sharp, intense t‐Se XRD reflections in the biofilm condition after only 168 h at 28°C without any thermal treatment implies that the EPS matrix substantially lowers the kinetic barrier to trigonal nucleation, effectively functioning as a crystallization catalyst or template operating at ambient temperature. This is, to our knowledge, the first reported study describing an ambient‐temperature, EPS‐driven crystallization of trigonal Se by a bacterial biofilm system, distinguishing this process mechanistically from the thermally driven recrystallization routes described elsewhere.

#### 3.1.2 HAADF‐STEM and SAED: Nanorod Morphology and High‐Resolution Crystallographic Evidence

HAADF‐STEM imaging revealed pronounced differences in the quantity, structure, morphology, spatial distribution, and aggregation state of biogenic Se nanoparticles (SeNPs) produced under the three physiological conditions, providing direct nanoscale visualization of the structural consequences of EPS templating (Figure S2). Biofilm‐associated cells generated a high density of elongated, anisotropic nanostructures distributed mainly in the extracellular space surrounding cell clusters. These structures ranged from 250 to 500 nm in length and exhibited a high aspect ratio characteristic of nanorod morphology (Figure S2A). Both the abundance and the degree of anisotropy of these nanorods were significantly higher in the biofilm state compared to SeNPs produced by planktonic or EPS‐depleted cells (Figure S2C,E). Furthermore, the nanorods demonstrated a pronounced tendency toward lateral aggregation and co‐alignment. This suggests that EPS functional groups not only template individual nanorod formation, but may also mediate secondary assembly into higher‐order nanorod bundles through surface‐directed interactions. Finally, EDX elemental mapping confirmed Se as the dominant elemental constituent of these structures (Figure S2B,D,F). We note that the present analysis characterizes the allotropic outcome qualitatively—through phase identity, morphology, and crystallographic orientation—rather than quantitatively; systematic determination of nanorod yield, size and aspect‐ratio distributions, and crystalline phase fractions (e.g., by Rietveld refinement) will be required to benchmark synthesis efficiency for process development.

**FIGURE 2:**
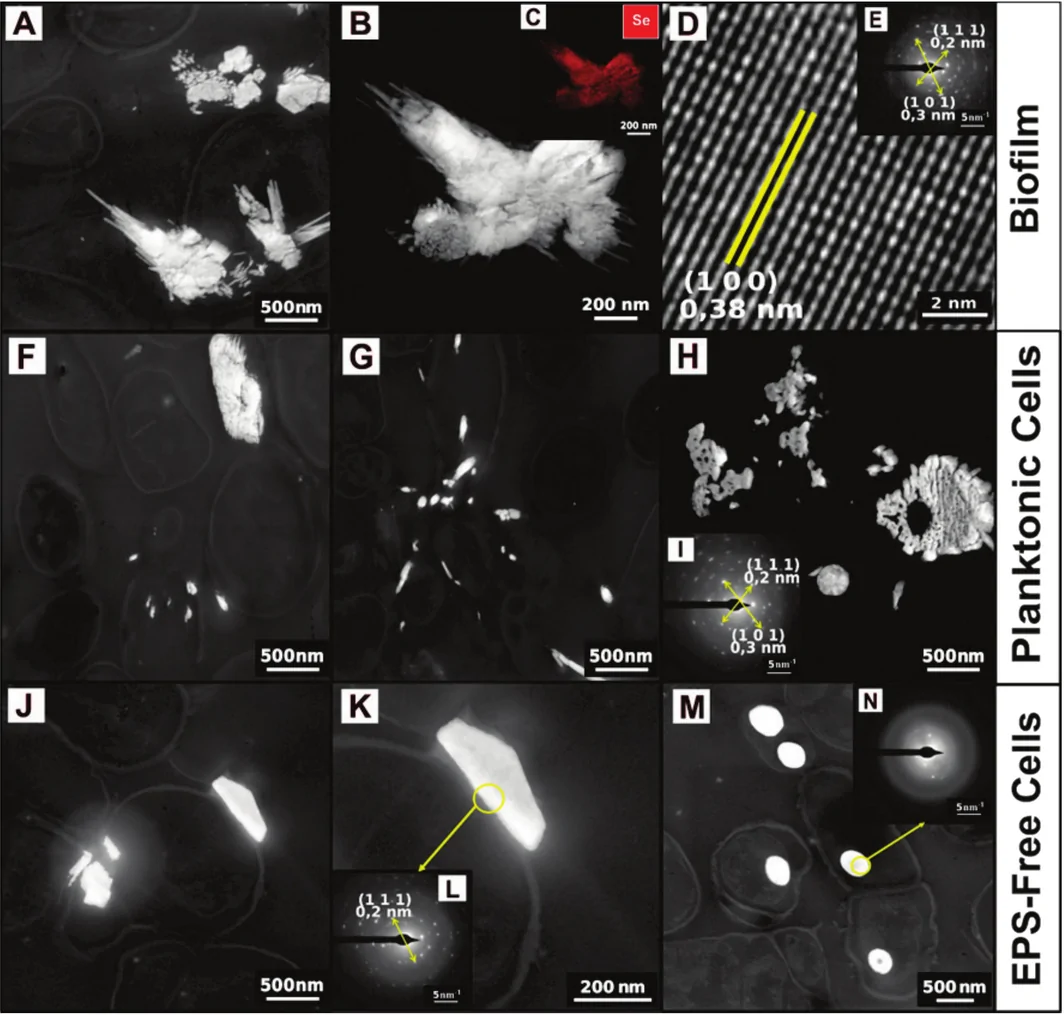
Close view of Se nanoparticles synthesized by different cellular states of *S. bentonitica*. High‐magnification HAADF‐STEM and HRTEM images of Se nanorods synthesized by biofilm‐associated *S. bentonitica* cells (A–C), EDX map (C), high‐resolution TEM with lattice spacings (D), and SAED pattern (E). Panels F–H show nanoparticles produced by planktonic cells, with SAED image (I) showing their diffraction pattern. Panels (J–M) depict nanoparticles from EPS‐free cells. SAED analysis (panels L and N) reveals the reduced number of crystalline nanostructures relative to biofilm‐derived samples.

High‐resolution TEM and SAED analysis of biofilm‐derived nanostructures provided atomic‐scale confirmation of crystallinity and crystallographic orientation (Figure 2A–E). The SAED pattern obtained from individual biofilm nanorods showed well‐resolved discrete diffraction spots rather than diffuse rings, indicating single‐crystal or highly textured polycrystalline material (Figure 2E). These spots were indexed to the (101) and (111) planes of t‐Se. Lattice‐fringe analysis in high‐resolution TEM images confirmed interplanar spacings of 0.38 nm associated with the (100) plane, in excellent agreement with the accepted crystallographic parameters of t‐Se (Figure 2D). Fast Fourier transform (FFT) of the high‐resolution images produced spot patterns fully consistent with the SAED data, confirming that the atomic‐scale periodicity is maintained coherently across the imaged nanorod regions.

A critical crystallographic divergence was observed when comparing the biofilm to the planktonic and EPS‐depleted conditions. While the (101) and (111) reflections were detectable in both planktonic samples (Figure 2I) and EPS‐free cells (Figure 2L,N), the (100) reflection was consistently identified only in biofilm‐derived nanorods (Figure 2D,E). In the Se crystal system, helical Se chains run parallel to the c‐axis (001), which constitutes the natural elongation direction of the crystal (Mayers and Xia 2002). The (100) plane in trigonal Se represents the lateral facets of the helical Se chain axis. The exclusive presence of a (100) reflection in the biofilm condition and specifically its registration in SAED indicates that nanorod elongation along these crystallographic directions occurs with a high degree of orientational order and requires the organized template provided by the EPS matrix. This level of anisotropic crystal growth requires a template that interacts asymmetrically with specific crystal faces, suppressing lateral growth while permitting or promoting axial elongation, a function that the organized, helical protein components of EPS (particularly flagellin subunits) are structurally well‐positioned to perform. The absence of (100) reflections in planktonic and EPS‐depleted particles suggests that reduction without an organized EPS template produces predominantly isotropic nanostructures with random crystallographic orientation limited to the most thermodynamically stable plane, explaining the spherical morphology and complete lack of sustained elongation observed by HAADF‐STEM (Figure 2F–H,J–M).

The extracellular localization of Se nanorods within the biofilm provides important mechanistic insights, contrasting sharply with the behaviour of spherical nanoparticles. Nanospheres localize primarily within the cytoplasm, an observation consistent with previous models of SeNP biosynthesis (Ruiz‐Fresneda et al. 2020) and particularly pronounced in EPS‐depleted samples (Figure 2M). This spatial segregation suggests a two‐stage biogenesis model: initial intracellular reduction of Se(IV) to Se(0) produces nanospheres at the site of enzymatic activity, consistent with the established intracellular selenite reduction pathways involving thioredoxin reductase, glutathione reductase, and sulfite reductase (Wang et al. 2022), followed by export of Se(0) intermediates or precursors into the extracellular EPS matrix. Once in the EPS environment, these materials come into contact with organized template structures, which are probably flagellin filaments and polysaccharide networks.

#### 3.1.3 FTIR Spectroscopy: Insights Into the Potential Role of EPS in Se(IV) Coordination and Reduction

**FIGURE 3:**
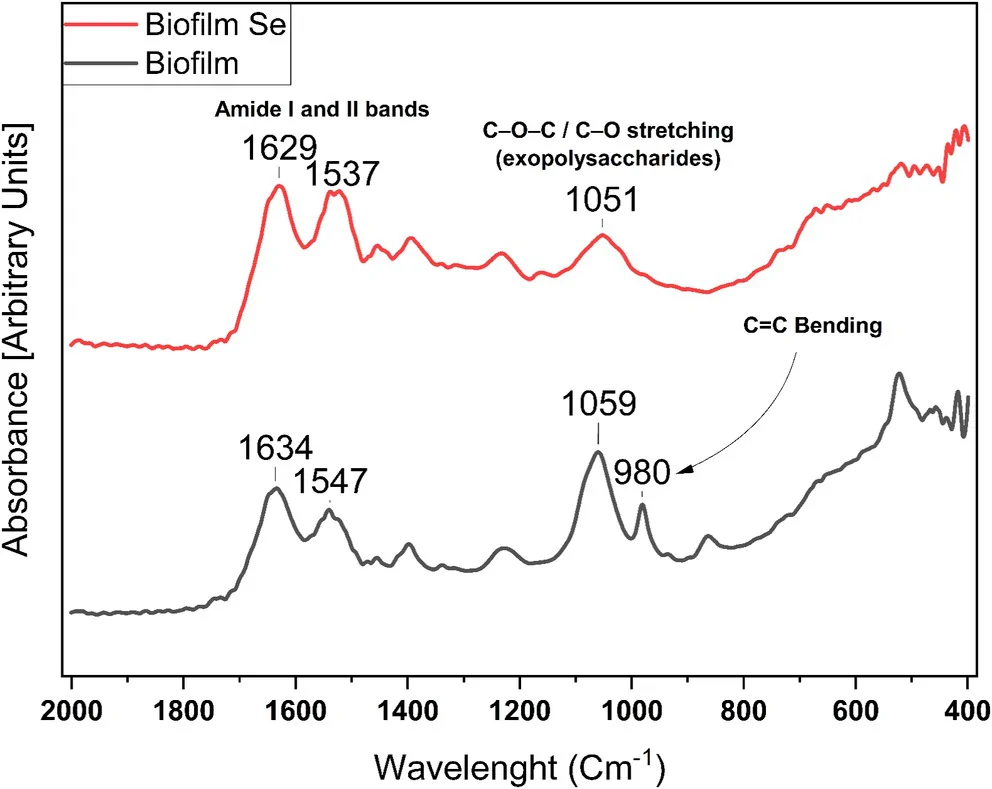
FTIR spectra of *Stenotrophomonas bentonitica* BII‐R7 biofilm‐associated samples formed in the absence (control) and presence of Se(IV). The spectra reveal notable shifts in key vibrational regions, including the amide I (~1650 cm^−1^) and amide II (~1540 cm^−1^) bands, indicative of protein conformational changes, as well as C–O–C stretching (~1200–1000 cm^−1^) and C=C bending (~980 cm^−1^) bands associated with polysaccharide rearrangements.

To investigate the molecular interactions through which EPS components participate in Se(IV) coordination and Se(0) crystallization, FTIR spectroscopy was performed on biofilm‐associated samples exposed to selenite, with comparison to control biofilms not exposed to Se(IV). The spectra revealed several significant band shifts and intensity changes that collectively implicate both the proteinaceous and polysaccharidic EPS fractions in direct chemical interaction with selenium species (Figure 3). In the protein‐dominated spectral region, pronounced shifts were observed in the amide I band (~1650 cm^−1^), which reports on C=O stretching of the peptide backbone and is sensitive to protein secondary structure, and in the amide II band (~1540 cm^−1^), reflecting N‐H bending and C‐N stretching vibrations. These shifts indicate conformational changes in EPS‐associated proteins upon selenium exposure either through direct coordination of selenium species to amino acid side chains (particularly cysteine thiol groups and lysine amine groups) or through indirect structural reorganization driven by Se‐induced changes in the local chemical environment.

In the polysaccharide‐dominated spectral region, modifications in C–O–C stretching vibrations (~1050–1150 cm^−1^) and C=C bending modes were consistent with structural rearrangements or chemical modification of the carbohydrate EPS fraction. Polysaccharide carboxyl groups (–COO^−^, ~1400 cm^−1^) and hydroxyl groups (–OH, ~3300 cm^−1^) are among the most important metal‐binding functional groups in bacterial EPS, and their spectroscopic perturbation by Se(IV) exposure suggests direct electrostatic coordination of selenite oxyanions to these sites. This interpretation is consistent with published FTIR studies of EPS‐metal interactions in other systems, where analogous band shifts have been attributed to the formation of inner‐sphere complexes between selenite and carboxylate or hydroxyl groups (Larson et al. 2023; Saikia et al. 2024).

The protein conformational changes are particularly significant given that flagellin, the principal structural subunit of bacterial flagella and a major EPS component, is markedly upregulated in BII‐R7 under Se(VI) stress (Ruiz‐Fresneda, Fernández‐Cantos, et al. 2023). Notably, flagellin from *Pseudomonas fluorescens* has been documented to template the formation of one‐dimensional silica nanotubes and Au(111) surfaces, demonstrating that the helical repeating structure of flagellin filaments can impose directional constraints on nanomaterial growth (Wang et al. 2008; González Orive et al. 2014). Taken together, the FTIR data suggest a model in which polysaccharide EPS components function primarily in Se(IV) ion capture and concentration through electrostatic coordination, while proteinaceous components, particularly flagellin, provide the structural template that imposes crystallographic directionality on Se(0) nucleation and elongation in the extracellular matrix space.

### 3.2 Quorum Sensing‐Independent Biofilm Formation Preserves EPS Functionality Under Metal Stress

The EPS‐mediated nanorod biosynthesis demonstrated here is contingent upon the maintenance of a structurally intact, EPS‐producing biofilm, a condition that must be preserved even under the metal stress conditions inherent to bioremediation settings. Heavy metals, including selenite, nickel, and cadmium, are known to suppress bacterial biofilm formation by disrupting the synthesis or signalling function of N‐acyl homoserine lactones (AHLs)—the central quorum sensing (QS) molecules that regulate EPS production and biofilm maturation in many species (Fechner et al. 2012; Huedo et al. 2018). We therefore investigated whether *S. bentonitica* BII‐R7 maintains its biofilm‐forming capacity under metal stress and whether any observed resilience could be explained by its genomic content.

**FIGURE 4:**
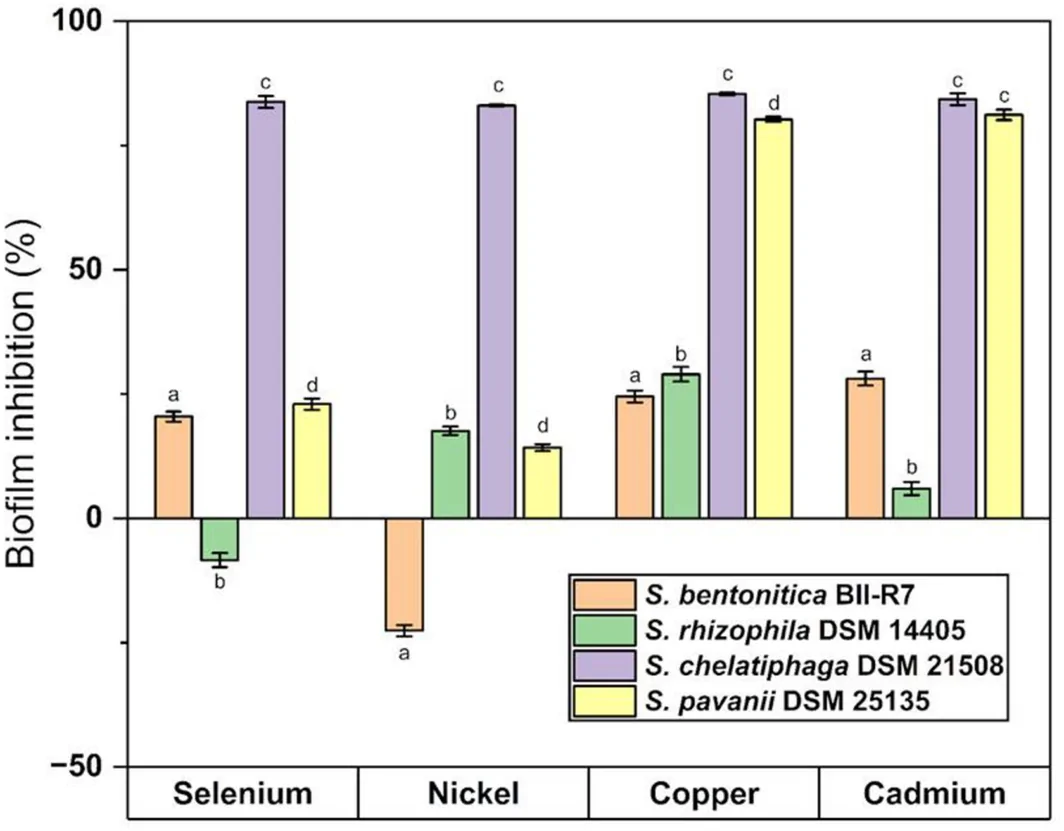
Biofilm formation assay in the four *Stenotrophomonas* strains. The assay was performed after 72 h of incubation. Bars represent the mean biofilm production ± standard deviation. Different letters above the bars indicate statistically significant differences among strains (*p* < 0.05, one‐way ANOVA followed by Tukey's post hoc test). The percentage of biofilm inhibition is indicated relative to the native biofilm formation (treatment without metals). Error bars represent the standard deviation from three independent biological replicates (*n* = 3).

Quantification of biofilm production by BII‐R7 and three environmental *Stenotrophomonas* species ( *S. chelatiphaga* DSM 21508, *S. pavanii* DSM 25135, and *S. rhizophila* DSM 14405) under control conditions and in the presence of 2 mM Se(IV), Cu(II), Ni(II), and Cd(II) revealed strikingly different responses (Figure 4). While BII‐R7 produced a modest baseline biofilm relative to the other strains under metal‐free conditions, it exhibited the greatest resilience to metal‐induced disruption across all four metals tested. Most remarkably, BII‐R7 biofilm biomass increased by 21.89% ± 2.52% in the presence of 2 mM Ni, indicating active metal‐stimulated biofilm enhancement rather than mere tolerance. In contrast, *S. chelatiphaga* DSM 21508 exhibited reductions exceeding 84% in biofilm production under all four metal stress conditions, *S. rhizophila* DSM 14405 showed reductions of 60%–75%, and *S. pavanii* DSM 25135 showed intermediate responses. The contrast between BII‐R7's metal‐resilient biofilm and the metal‐sensitive biofilms of the other strains is central to the selenium nanorod synthesis phenotype. Only BII‐R7 maintains functional, EPS‐producing biofilms at the metal concentrations required for effective bioremediation. This may contribute to why EPS‐templated crystalline Se nanorod formation was observed only in this strain, if the metal‐sensitive biofilms of the other strains cannot sustain the structural continuity required for long‐range crystalline growth; this interpretation remains to be tested directly.

The metal‐responsive biofilm behaviour of BII‐R7 is notable when placed against the recent literature. In most bacteria, sub‐lethal concentrations of copper, nickel, and cadmium suppress biofilm formation: nickel, for example, shuts down manganese oxidation and pellicle biofilm formation in *Pseudomonas putida* GB‐1 (Marques Mendonca et al. 2023), and copper and cadmium disrupt quorum‐sensing‐dependent biofilm maturation across diverse taxa (Fechner et al. 2012; Recalde et al. 2023). The metal‐stimulated biofilm enhancement observed here for BII‐R7 (a 21.9% increase under 2 mM Ni) therefore runs counter to the general trend and is, to our knowledge, unusual among environmental *Stenotrophomonas* isolates. This resilience is also directly relevant to the growing interest in microbial and biofilm‐mediated routes to functional nanomaterials, in which the extracellular matrix can serve as both a reducing and a structuring environment for metal and metalloid nanoparticles (Belew et al. 2025); a biofilm that remains intact under polymetallic stress is a prerequisite for exploiting such extracellular, matrix‐templated synthesis in real contaminated settings.

Comparative analysis of biofilm‐regulatory genes across the four strains revealed a striking genomic explanation for BII‐R7's exceptional biofilm resilience. Screening for genes involved in quorum sensing (*rpfF, ax21, smoR*), polysaccharide biosynthesis (*spgM, rmlA, rmlC*), and flagella assembly (*fleQ, flgE/G/K/I, flhA, fliF/I/K/M/N/O/A, fimV*) showed that BII‐R7 is the only strain in this comparison that lacks both *rpfF* and *smoR*, the two central regulators of the *Stenotrophomonas* diffusible signal factor (DSF) quorum‐sensing system (Figure S3). The *rpfF* gene encodes the DSF synthase responsible for producing the diffusible signal fatty acid that coordinates density‐dependent biofilm regulation, while *smoR* encodes the cognate transcriptional regulator. All three strains possess at least one of these genes, implicating a functional QS‐dependent biofilm regulation mechanism in each. The consequences of QS‐dependent versus QS‐independent biofilm regulation under metal stress are explained by the mechanism of metal toxicity on QS systems: metals such as Ni and Cd complex with AHL‐like signalling molecules and inactivate sensor kinases, thereby blocking DSF‐mediated biofilm induction in QS‐dependent strains (Fechner et al. 2012; Recalde et al. 2023). By bypassing AHL‐mediated signalling, BII‐R7 is expected to be largely insensitive to this metal‐induced QS disruption. The modest baseline biofilm production by BII‐R7 reflects the metabolic economy of a simpler regulatory architecture (Figure S4). In the nutrient‐sparse, bentonite clay environment from which BII‐R7 was isolated, this trade‐off is advantageous: eliminating energy‐costly QS‐dependent biofilm maturation redirects metabolic resources toward metal efflux, antioxidant defence, and other stress responses, while preserving the basal EPS‐producing capacity required for metal sequestration and nanoparticle templating. This is consistent with adaptive simplification observed in other oligotrophic bacteria that maintain functional but metabolically economical biofilms under resource limitation (Maughan and Redfield 2009).

The ecological contexts of the comparator strains further illuminate why QS‐dependent regulation is appropriate in some niches but not in metal‐contaminated environments. *S. chelatiphaga* DSM 21508, isolated from nutrient‐rich sewage sludge, likely benefits from QS‐coordinated biofilm dominance against intense microbial competition. *S. pavanii* DSM 25135 and *S. rhizophila* DSM 14405, both plant‐associated, plausibly rely on QS for root colonization and plant‐microbe signalling in dynamic rhizosphere environments where cell–cell communication confers clear ecological advantages (Xiong et al. 2020). The QS‐independent strategy of BII‐R7 is therefore not generally superior but is specifically adapted to oligotrophic, metal‐contaminated environments where QS disruption by heavy metals would be lethal to biofilm maintenance. This niche‐specific adaptation has the practical consequence of positioning BII‐R7's biofilm as uniquely stable under precisely the conditions encountered in seleniferous‐contaminated environments, copper‐contaminated soils, and mixed heavy metal settings.

### 3.3 Identification of Exclusive Genes Underpinning BII‐R7's Multimetal Resilience and EPS‐Remodelling Capacity

*S. bentonitica* BII‐R7 has now demonstrated two exceptional and interconnected capacities: metal‐resilient, QS‐independent biofilm formation and EPS‐templated biosynthesis of crystalline t‐Se nanorods that no other tested strain can replicate. BII‐R7 emerges as a promising chassis for green nanotechnology. Its unique profile, combining nonpathogenic safety, environmental robustness, and the rare ability to template crystalline t − Se nanorods, bridges the gap between laboratory biotransformation and industrial resource recovery. By leveraging its quorum‐sensing‐independent biofilm resilience, BII‐R7 can transform toxic selenite into a settleable, semiconducting product, offering a potential sustainable alternative to conventional chemical synthesis. Comparative genomics was therefore deployed not merely to explain known phenotypes but as a discovery tool: given BII‐R7's demonstrated exceptionality, comparative genomics was used to identify what further capacities encoded in its exclusive genome may be exploited for biotechnological or environmental applications. A panel of 38 *Stenotrophomonas* genomes was used for this purpose (Table S1). The re‐sequenced BII‐R7 genome (4,398,470 bp across 29 contigs; N50 of 327,036 bp; 99.12% completeness) represents a major quality improvement over the previous draft assembly (N50 of 35,432 bp, 94.28% completeness; Table S2) and provided a reliable basis for comparative analysis (BioProject PRJNA1232115).

#### 3.3.1 Pangenome Structure Contextualizes BII‐R7 as an Outlier in Exclusive Gene Content

Pangenome analysis using Roary v3.12.0 (with a sequence identity threshold of 90%) across the 38 genomes found a total of 26,068 gene clusters divided into four groups: core genome (670 genes, found in all 38 strains), soft‐core (73 genes, found in 37 strains), shell (3766 genes, found in 5–36 strains), and cloud genome (21,559 genes). Average Nucleotide Identity (ANI) analysis confirmed the taxonomic affiliations and highlighted the genomic divergence of BII‐R7 from the rest of the analysed strains (Figure S5). The small core genome and expansive cloud genome define an open pangenome structure, indicating ongoing gene gain and loss across the genus, a pattern consistent with the ecological versatility and frequent horizontal gene transfer (HGT) events characteristic of *Stenotrophomonas* (Ryan et al. 2009) (Figure S6 and Table S3). Importantly, the absence of plasmid DNA in all 38 genomes establishes that metal resistance and biofilm traits are chromosomally encoded in this genus, meaning that these adaptive capacities are stable and heritable rather than dependent on the transfer or loss of mobile genetic elements (Ochoa‐Sánchez and Vinuesa 2017).

**FIGURE 5:**
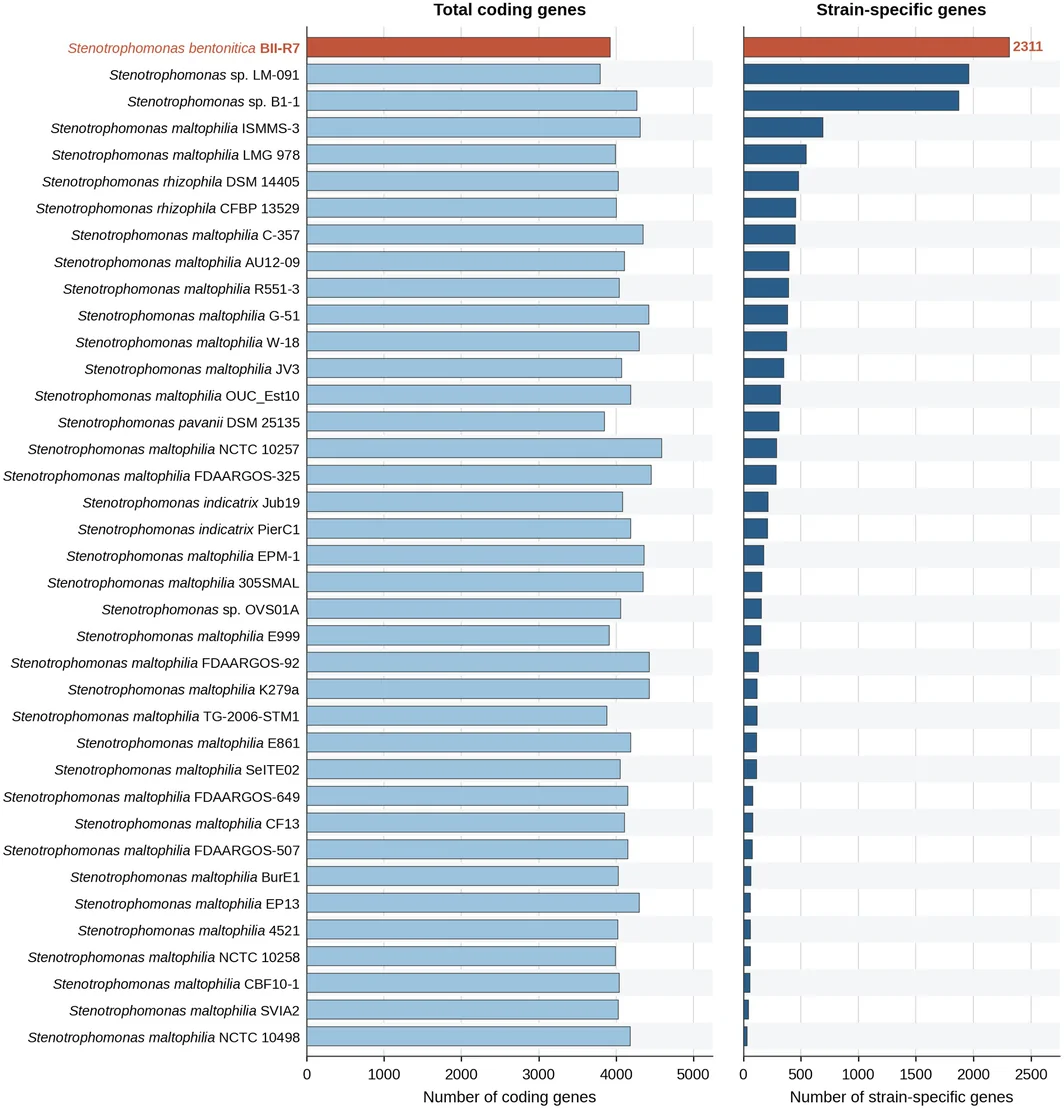
Diagram showing the number of genes coded by each strain and unique genes across all strains. The left bars show the number of coding genes found in each *Stenotrophomonas* strain included in the analysis, while the right bar graph shows the number of unique genes detected across all the studied strains.

The pangenome matrix and core‐genome phylogenetic tree highlighted the extensive accessory genome and unique evolutionary position of BII‐R7 within the genus (Figure S7). It harbours over 2300 exclusive genes. Environmental strains, particularly soil‐derived isolates, carry more unique genes than clinical *S. maltophilia* strains, consistent with the greater functional challenges of heterogeneous, nutrient‐variable, metal‐contaminated soil environments compared with the relatively stable conditions of clinical settings (Figure 5). The BII‐R7 exclusive genes show sequence homology with diverse soil and aquatic bacterial taxa, including *Pseudomonas citronellolis* , *P. aeruginosa* , *Parazoarcus communis*, *Xanthomonas* spp., and members of the Bradyrhizobiaceae, suggesting multiple independent HGT events from functionally diverse environmental donors, a pattern consistent with long‐term residence in geochemically complex bentonite clay formations. Rather than surveying all 2300 exclusive genes in detail, we focused specifically on those with experimentally validated or strongly predicted functions in metal resistance and biofilm regulation, as identified by BLASTx comparison against the BacMet database (*E*‐value < 0.01; sequence similarity > 75%; alignment coverage > 80%).

#### 3.3.2 Exclusive EPS‐Biosynthesis and Remodelling Genes as Structural Determinants of the Nanorod‐Templating Phenotype

**FIGURE 6:**
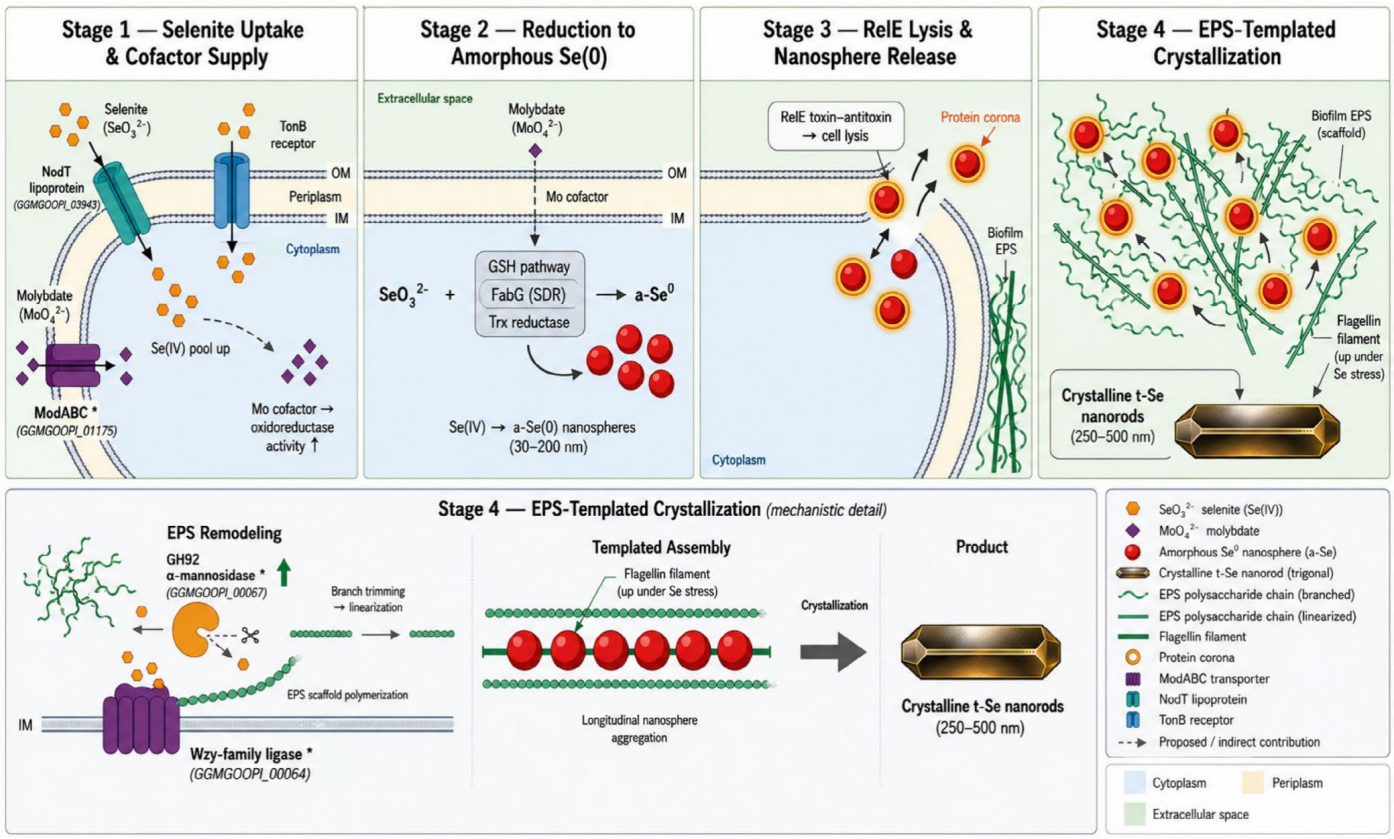
Proposed four‐stage model for EPS‐templated biosynthesis of crystalline trigonal selenium nanorods in *Stenotrophomonas bentonitica* BII‐R7. Stage 1: Selenite (SeO₃^2−^) is taken up through NodT (GGMGOOPI_03943) and TonB‐dependent receptors, while ModABC (GGMGOOPI_01175) imports molybdate (MoO₄^2−^), supporting reductive enzymes. Stage 2: Cytoplasmic oxidoreductases (glutathione pathway, FabG, thioredoxin reductase) reduce Se(IV) to amorphous Se(0) nanospheres (30–200 nm). Stage 3: Nanosphere accumulation activates the RelE toxin–antitoxin system, causing localized lysis and nanosphere release with a protein corona. Stage 4: Released nanospheres interact with biofilm EPS, where upregulated GH92 α‐mannosidase (GGMGOOPI_00067), Wzy‐family ligase (GGMGOOPI_00064), and flagellin filaments promote scaffold remodelling, longitudinal aggregation, and crystallization into trigonal selenium nanorods (250–500 nm). Dashed arrows indicate proposed contributions.

Among the 2300 exclusive genes identified in the BII‐R7 accessory genome, two singletons, GGMGOOPI_00064 and GGMGOOPI_00067, are the only EPS‐biosynthesis and remodelling proteins exclusive to BII‐R7 across the entire 38‐strain pangenome. These genes provide the strongest genomic and structural rationale for the nanorod‐templating phenotype, although definitive functional attribution awaits targeted mutagenesis. To evaluate this hypothesis, we performed integrated topological and structural modelling of both proteins and situated their predicted functions within a four‐stage mechanistic model for the EPS‐templated biosynthesis of crystalline *t*‐Se nanostructures, synthesizing the XRD, HAADF‐STEM, SAED, ATR‐FTIR, and pangenomic evidence reported here with prior mechanistic work on *S. bentonitica* BII‐R7 (Ruiz‐Fresneda et al. 2018, Ruiz‐Fresneda, Fernández‐Cantos, et al. 2023; Pinel‐Cabello et al. 2021) (Figure 6). In the first stage, Se(IV) is taken up through outer membrane transporters, including RND‐family lipoproteins (NodT), TonB‐dependent receptors, and metal cation transporters, concentrating selenite within the periplasm and cytoplasm (Ruiz‐Fresneda, Martinez‐Moreno, et al. 2023). The exclusive ModABC molybdate transporter (GGMGOOPI_01175) provides an additional high‐affinity uptake route for oxyanions of comparable tetrahedral geometry to molybdate, further elevating the intracellular Se(IV) pool available for subsequent reduction (Figure 6, Stage 1). In the second stage, cytoplasmic oxidoreductases, principally through the glutathione pathway, the SDR‐family reductase FabG, and thioredoxin‐disulfide reductase, reduce Se(IV) to amorphous Se(0) nanospheres (30–200 nm), as directly visualized by HAADF‐STEM at 24 h (Ruiz‐Fresneda et al. 2018) (Figure 6, Stage 2). In the third stage, intracellular nanoparticle accumulation triggers programmed cell death via the RelE toxin‐antitoxin system, releasing amorphous Se(0) nanospheres through lysis, a mechanism supported by the spatial co‐localization of lysed cells with extracellular particles and the complete absence of membrane vesicles in HAADF‐STEM images (Ruiz‐Fresneda et al. 2018) (Figure 6, Stage 3).

In the fourth and mechanistically decisive stage, released nanospheres encounter the EPS matrix of the biofilm, where the intact extracellular scaffold would promote the formation of crystalline nanorods. Two non‐mutually exclusive interactions may operate at this stage. First, amorphous Se(0) nanospheres released into the extracellular space associate with EPS polysaccharide chains and flagellin filaments; the latter are upregulated under Se stress and established templates for one‐dimensional nanostructure growth (Ruiz‐Fresneda et al. 2018), providing the structural axis along which nanospheres coalesce and undergo the stepwise allotropic transition from amorphous Se(0) through a monoclinic intermediate to thermodynamically stable *t*‐Se. Second, our ATR‐FTIR data demonstrate perturbations of EPS carboxylate and hydroxyl stretching frequencies under selenite stress, consistent with direct Se(0)NPs–EPS coordination, which raises the possibility that a small fraction of Se(IV) is concentrated and reduced within the EPS matrix itself rather than exclusively within the cell. Whether the primary substrate for EPS‐templated crystallization is pre‐formed Se(0) nanospheres or EPS‐associated Se(IV) undergoing extracellular reduction cannot be resolved from the present data and warrants further investigation. The critical role of EPS architecture in this process is demonstrated by two complementary lines of evidence. First, the characteristic (100) and (101) diffraction planes of the t‐Se lattice were detected mainly in biofilm‐derived samples and were absent in EPS‐depleted controls, even though the latter retained residual XRD signal consistent with minor amorphous Se(0) accumulation. Second, the diverse nanorod morphologies, nanowires, hexagonal plates, and polygonal nanoforms of 30–400 nm, confirmed by HAADF‐STEM and SAED in biofilm samples, were markedly reduced under EPS depletion, although not entirely absent. Taken together, these observations indicate that intact EPS architecture is the predominant determinant of efficient crystalline nanorod formation in BII‐R7, while residual crystallization can occur to a minor extent in its absence.

The molecular basis for this uniquely anisotropic outcome is most parsimoniously attributed to two exclusive EPS‐biosynthesis and remodelling proteins encoded in the BII‐R7 accessory genome, whose cooperative function is illustrated in Figure 6, Stage 4. These are the only two EPS‐related proteins exclusive to BII‐R7 across the entire 38‐strain pangenome for which a direct structural and functional explanation of nanorod templating can be proposed. The first candidate, GGMGOOPI_00064, is a Wzy‐family polysaccharide ligase. Topology prediction using DeepTMHMM (Hallgren et al. 2022) unambiguously assigned 12 transmembrane α‐helices with an intracellular N‐terminus (posterior probability ≈1.0 per helix), validating the canonical Wzy fold and consistent with O‐antigen ligation activity at the periplasmic face, independently of structural modelling. AlphaFold3 modelling yielded high global confidence (pTM = 0.89; mean pLDDT = 89.67), and residue‐level confidence parsing revealed that the 14%–28% sequence divergence relative to Wzy homologues across all comparator strains is concentrated exclusively in the extracellular loop domain, the region that directly contacts the growing O‐antigen chain and governs ligation geometry in all Wzy‐family enzymes (Whitfield 2006). Critically, the divergent fifth extracellular loop (EC5; residues 281–371) adopts a fully defined three‐dimensional structure (mean pLDDT = 85.6). This 91‐residue structured domain is enriched in metal‐coordinating residues, 12 acidic residues (Asp, Glu), 3 histidines, and 2 cysteines, whose carboxylate, imidazole, and thiolate side‐chains are established inner‐sphere ligands for selenium oxyanions (Saikia et al. 2024; Figure 6). We propose that the divergent EC5 loop governs the stereochemical configuration of inter‐repeat unit glycosidic linkages during O‐antigen/EPS polymerization at the periplasmic face of the inner membrane, consistent with its topological localization. The net consequence is a secreted polysaccharide scaffold with a charge distribution, chain rigidity, and metal‐chelating capacity that is structurally and compositionally unlike the EPS of any other tested *Stenotrophomonas* strain. The ATR‐FTIR‐detected perturbations of carboxylate stretching frequencies under Se(IV) stress are consistent with Se(IV)–carboxylate interactions at the EPS interface, though the precise coordination mode cannot be resolved from FTIR data alone. This divergence is mechanistically meaningful: recent structural and computational studies of Wzy polymerases confirm that the sequence and geometry of extracellular loops directly determine the stereochemical configuration (α‐ or β‐glycosidic bond) of inter‐repeat unit linkages, providing the molecular logic by which a divergent EC5 in BII‐R7 would produce a polysaccharide scaffold with distinct spacing, charge density, and metal‐coordination geometry relative to all comparator strains (Ganaie et al. 2025).

The second candidate, GGMGOOPI_00067, is a GH92‐family α‐mannosidase. DeepTMHMM confirmed a globular topology preceded by a signal peptide (residues 1–27), localizing the mature enzyme to the extracellular space where nanorod assembly occurs (Figure 6, Stage 4). BLASTP identifies its closest homologues (83%–85% identity) in *S. rhizophila* , *S. indicatrix*, *S. lactitubi*, *S. cyclobalanopsidis*, and *S. hibiscicola*, all below the 90% Roary exclusivity threshold. AlphaFold3 modelling of the 1082‐residue mature domain yielded exceptional confidence (pTM = 0.95). The 15%–17% sequence divergence from homologues is concentrated in the substrate‐binding loop regions governing linkage selectivity in the GH92 family (Zhu et al. 2010), predicting altered cleavage specificity and a mannose linkage pattern in BII‐R7 EPS distinct from that of all comparator strains. By enzymatically trimming α‐mannosyl side‐branches from the nascent polysaccharide, the enzyme is predicted to reduce steric hindrance, linearize EPS fibrils, and transform a disordered carbohydrate mesh into a high‐density scaffold of parallel nano‐channels of uniform geometry. Crucially, GGMGOOPI_00067 is transcriptionally induced under selenium stress (Pinel‐Cabello et al. 2023; Ruiz‐Fresneda, Fernández‐Cantos, et al. 2023), consistent with its proposed role in assembling the biomineralization template in temporal and spatial coincidence with Se(0) nanosphere export. This prediction is grounded in a metadynamics study of bacterial GH92 α‐1,2‐mannosidases which resolved the catalytic mechanism at atomic resolution, demonstrating that a Ca^2+^ ion distorts the mannose ring into a boat conformation for nucleophilic attack by catalytic Asp and Glu residues and that the substrate‐binding loops flanking the active site are the primary structural determinants of α‐mannosyl linkage selectivity (Kołaczkowski et al. 2023). Because the sequence divergence in BII‐R7 GGMGOOPI_00067 is concentrated precisely in these substrate‐binding loops, the prediction of altered cleavage specificity has independent support beyond structural modelling, reinforcing the case that this enzyme produces a compositionally and structurally unique EPS glycan architecture with enhanced capacity to coordinate and template Se(0) precursors.

We propose that the cooperative action of these two exclusive proteins produces a secreted EPS scaffold that is compositionally and structurally different in the genus. Specifically, the divergent EC5 loop of the Wzy‐family ligase generates a polysaccharide repeat unit with a unique charge distribution and metal‐coordination geometry at the periplasm–EPS interface, while the secreted GH92 α‐mannosidase reduces the steric bulk of the maturing EPS by trimming α‐mannosyl branches, collectively increasing the density and accessibility of metal‐chelating functional groups on the EPS backbone. We propose that this remodelled matrix provides the anisotropic chemical and structural environment, organized along upregulated flagellin filaments, that templates the longitudinal aggregation of amorphous Se(0) nanospheres and their transition to crystalline *t*‐Se. This is consistent with the exclusive detection of (100) XRD reflections in biofilm‐associated samples. Several *Stenotrophomonas* strains, particularly *S. maltophilia* , have previously been reported to reduce selenite/selenate to elemental selenium and produce Se nanoparticles (Dungan et al. 2003; Di Gregorio et al. 2005; Lampis et al. 2017). However, no equivalent biofilm‐associated formation of crystalline trigonal Se nanorods has, to our knowledge, been described in the genus under comparable conditions. Direct causal attribution of this phenotype to these two loci will require targeted knockout and complementation experiments. We further note that factors beyond EPS composition—including intracellular redox balance, the kinetics of Se(IV) reduction, the activity of dedicated selenium reductases, and local supersaturation within the matrix—may modulate the amorphous‐to‐trigonal transition. The present data cannot exclude these contributions, and disentangling them from the proposed EPS‐templating mechanism will require the targeted genetic and biochemical dissection outlined below.

This represents, to our knowledge, the first genomics‐ and structural‐modelling‐informed mechanistic framework, grounded in direct phenotypic evidence, for ambient‐temperature EPS‐driven biosynthesis of crystalline *t*‐Se nanorods by a bacterial biofilm. Studies on selenite biotransformation by *Bacillus mycoides* , *Pseudomonas putida* , and *Escherichia coli* have reported amorphous spherical Se(0) nanoparticles under planktonic or cell‐free conditions, without evidence of EPS‐driven crystalline nanostructure formation (Kora and Rastogi 2016; Lampis et al. 2014; Debieux et al. 2011), although systematic comparisons under biofilm conditions have not been reported. The clear spatial segregation of intracellular nanosphere formation from extracellular nanorod assembly establishes that the crystalline phenotype is fundamentally an EPS‐promoted extracellular phenomenon; nanostructures appearing to overlap with the cellular boundary in two‐dimensional micrographs (Figure 2F) are interpreted as cell surface‐associated or tightly bound EPS‐embedded structures, consistent with the active export of Se(0) nuclei to the organized biofilm matrix where final anisotropic growth occurs (Figure 2A,B). The closest mechanistic analogy in the broader biomineralization literature is the EPS‐mediated transformation of amorphous calcium carbonate into monohydrocalcite and calcite in tufa‐depositing biofilms (Broughton 2023), in which polysaccharide and protein EPS components jointly direct polymorph selection and crystal morphology. The present study extends this class of EPS‐driven biomineralization to a transition‐element chalcogenide for the first time and identifies accessory genome evolution as a potential mechanism by which bacteria acquire the capacity to program mineral morphology through targeted EPS remodelling.

This finding holds considerable potential for industrial applications. In selenium bioremediation, the preferential production of *t*‐Se nanorods confers markedly enhanced settleability and filterability, translating directly into improved operational efficiency of bioreactor‐based selenium removal systems treating mining drainage, coal combustion effluents, and agricultural runoff (Dessì et al. 2016; Ojeda et al. 2020). In green nanotechnology, the biofilm‐based route described here provides a scalable, low‐energy, ambient‐temperature alternative to conventional physicochemical synthesis, which requires high temperatures, toxic reducing agents, or organic solvents; the photoconductive, semiconducting, and spectral sensitivity properties of *t*‐Se nanorods make them directly applicable in solar cell fabrication, xerographic systems, photodetectors, and rectifiers (Ibragimov et al. 2000). In biomedical nanotechnology, crystalline Se nanostructures have been reported to exhibit superior antitumoral and antimicrobial activity relative to amorphous counterparts, attributed to their greater structural integrity and more defined surface chemistry (Kuršvietienė et al. 2020; Filipović et al. 2021). Translational evidence for the practical applicability of BII‐R7‐based biotransformation in engineered systems is provided by González‐Morales et al. (2025), who demonstrated up to 70% Se(IV) removal from contaminated aqueous solutions using BII‐R7 immobilized in alginate hydrogels; given the present findings, promoting biofilm development within such matrices is predicted to further enhance removal efficiency and enable selective recovery of crystalline *t*‐Se nanorods with defined morphology.

### 3.4 Exclusive Metal Resistance Determinants Explain BII‐R7's Superior Multimetal Tolerance

In‐depth analysis of BII‐R7's exclusive gene set against the BacMet database identified a suite of candidate metal resistance genes absent from all 37 comparator genomes, providing a genomic explanation for the strain's demonstrated ability to tolerate and transform selenium, uranium, curium, europium, copper, and nickel (Table S4). Among the most biotechnologically significant are two copper resistance determinants: *copF* (90.5% sequence identity; *E*‐value 0; GGMGOOPI_00882), encoding a copper‐translocating P‐type ATPase homologous to the CPX‐type copper exporters characterized in the extremophilic archaeon *Ferroplasma acidarmanus* and in *Sulfolobus solfataricus* (Baker‐Austin et al. 2005; Deigweiher et al. 2004), and *copB* (82.8% identity; *E*‐value 1.53 × 10^−165^; GGMGOOPI_01395), encoding a copper resistance protein. The co‐occurrence of *copF* and *copB* exclusively in BII‐R7, neither of which is present in any of the 37 genomes, suggests a capacity for copper detoxification via dual efflux mechanisms that the other strains lack.

Also exclusive to BII‐R7 are two genes encoding subunits of a molybdate ABC transporter, an ATPase subunit of the ModABC complex (GGMGOOPI_01175; 75.3% identity to tungsten/molybdenum resistance determinants) and the corresponding inner membrane subunit (GGMGOOPI_01176; 100% identity; *E*‐value 6.4 × 10^−120^). The functional relevance of molybdenum acquisition extends beyond primary metabolism: molybdopterin‐containing enzymes are directly implicated in selenate and selenite reduction in multiple bacterial systems, and molybdate ABC transporters have been shown to enhance molybdopterin cofactor availability, thereby supporting the reductive detoxification of Se oxyanions (Castro et al. 2020; Lashani et al. 2023). This genomic feature is consistent with previous transcriptomic data from BII‐R7 showing highly elevated expression of molybdopterin biosynthesis genes (*moeA*, 58‐fold; *moaE*, 56‐fold; *moaC/B*, 31‐fold) and the respiratory nitrate reductase beta subunit (180‐fold) under Se(VI) stress (Pinel‐Cabello et al. 2023), suggesting that Se reduction in BII‐R7 proceeds partly through a molybdenum‐dependent enzymatic pathway augmented by exclusive molybdate transport capacity. Transcriptomic profiling under metal stress confirmed the active transcription and upregulation of these exclusive resistance determinants in response to Se(VI) exposure (Figure S8). Additional exclusive genes include a NodT‐family RND efflux system outer membrane lipoprotein (GGMGOOPI_03943; 100% identity) and its cognate MarR‐type regulatory gene *emrR* (GGMGOOPI_03944), whose functional class (RND efflux) was previously identified as transcriptionally dominant under Se(VI) stress in BII‐R7 (133‐fold upregulation of a NodT‐family protein; Pinel‐Cabello et al. 2021), reinforcing the relevance of these exclusive determinants. Together, the exclusive multidrug/metal efflux pumps *smeF* (100% identity) and *smrA* (79.1% identity) provide additional efflux capacity. The convergence of genomic prediction, prior transcriptomic data, and current phenotypic data presents a coherent and well‐supported model in which BII‐R7's exceptional multimetal tolerance arises from an exclusive genetic repertoire encoding complementary, metal‐specific resistance mechanisms. The practical significance of this exclusive resistance arsenal extends beyond laboratory tolerance assays. Seleniferous contamination in real environments consistently presents as a polymetallic challenge: copper, nickel, zinc, and cadmium co‐occur with selenium oxyanions at concentrations that suppress biofilm formation in susceptible organisms (Tan et al. 2016; Dessì et al. 2016). Without the copF/copB copper efflux system, czcA‐mediated broad‐spectrum divalent cation extrusion and the molybdate import capacity that sustains selenate/selenite reduction, BII‐R7 would lose biofilm integrity under precisely the conditions relevant to bioremediation and, with it, the EPS scaffold demonstrated to be required for crystalline nanorod production. The exclusive resistance repertoire therefore functions as an enabling layer for the biotechnological phenotype: it is not metal tolerance as an end in itself but metal tolerance as the prerequisite for EPS‐maintained, nanorod‐templating biofilm function in multicontaminant matrices.

### 3.5 Functional Validation: Metal Tolerance Phenotypes and Gene Expression Confirm Genomic Predictions

To confirm that the genomic predictions of enhanced metal resistance translate to measurable phenotypes, growth curve analyses under graded Cu(II) and Ni(II) stress and RT‐qPCR quantification of exclusive resistance gene expression were performed using BII‐R7 and three comparator strains as a functional reference frame. It should be noted that these experiments were conducted under planktonic conditions and are therefore intended as a first‐order functional validation of genomic content rather than a direct reflection of the EPS‐associated physiology that is the central focus of this study.

**FIGURE 7:**
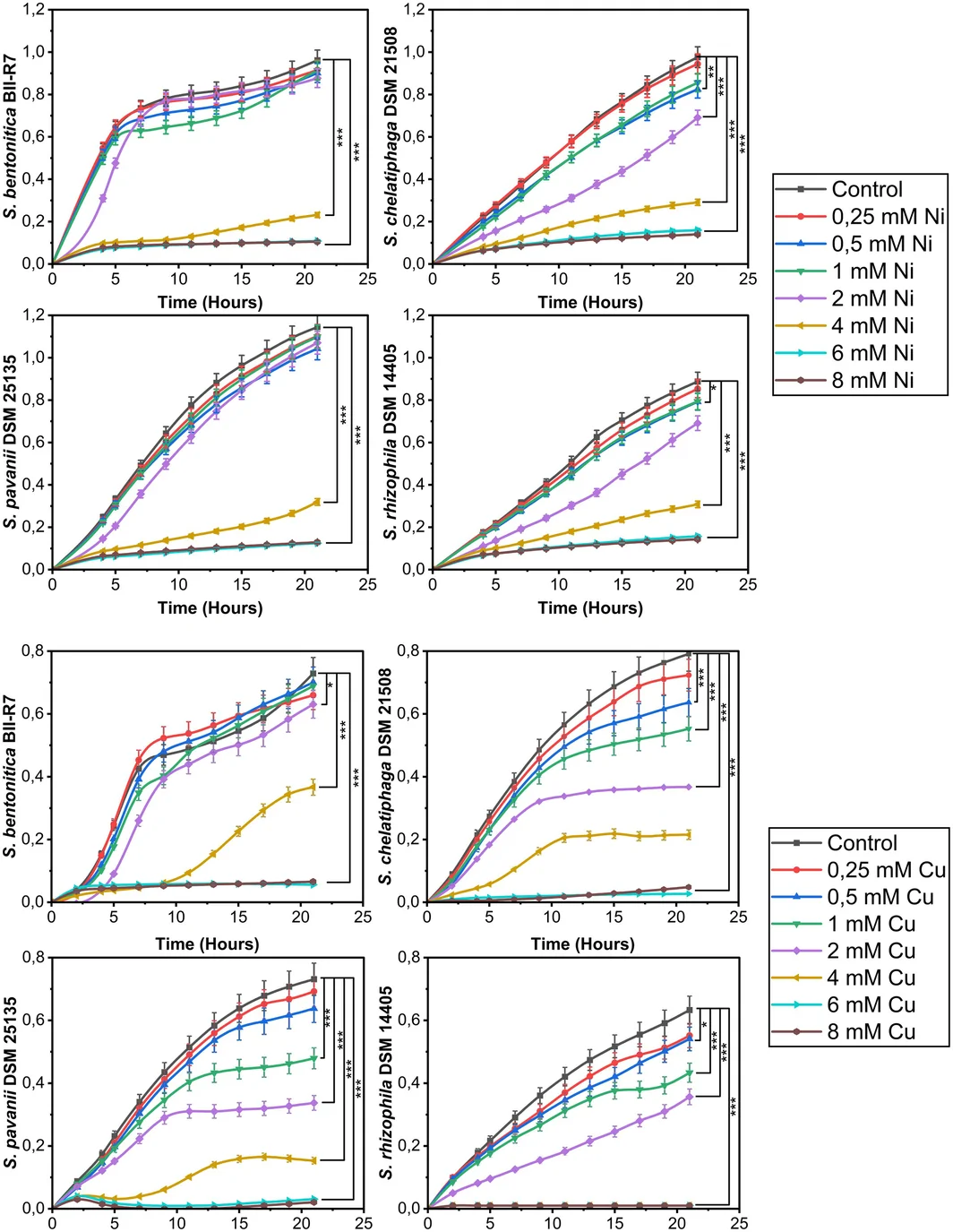
Growth curves of selected strains exposed to varying concentrations of copper (Cu) and nickel (Ni). Growth is represented as the relative increase in optical density (OD_600_) over time, normalized to the initial absorbance values. Statistical significance was assessed using one‐way ANOVA for each time point. Asterisks indicate statistically significant differences in growth relative to the control group: *p < 0.05, **p < 0.01, ***p < 0.001. Error bars represent the standard deviation from three independent biological replicates (*n* = 3).

Under graded Cu(II) stress (0–8 mM), BII‐R7 maintained growth comparable to the control at 2 mM Cu (OD_600_≈0.6), while all three comparator strains showed approximately 50% growth suppression at this concentration (OD_600_≈0.3; Figure 7), consistent with the exclusive *copF* and *copB* determinants identified in the BII‐R7 pangenome. Under graded Ni(II) stress (0–8 mM), BII‐R7 and *S. pavanii* DSM 25135 showed comparable superior tolerance at 2 mM Ni, while *S. chelatiphaga* DSM 21508 and *S. rhizophila* DSM 14405 showed pronounced inhibition (Figure 7), consistent with *czcA*‐mediated divalent cation efflux in BII‐R7. The similar Ni tolerance of *S. pavanii* in the absence of *czcA* or comparable efflux genes suggests that convergent tolerance can arise through distinct molecular mechanisms, underscoring the value of functional assays in qualifying genomic inference.

RT‐qPCR confirmed that the exclusive resistance genes are transcriptionally active and metal‐responsive: *copF* and *copB* were induced 8.6‐fold (*p* < 0.05) and 3.4‐fold (*p* < 0.05), respectively, under Cu(II) stress, and *czcA* was induced 42.2‐fold (*p* < 0.01) under Ni(II) stress, establishing these loci as active, regulated components of BII‐R7's adaptive metal response rather than cryptic or vestigial elements (Figure S9 and Table S5).

Taken together, the results support an integrated model in which BII‐R7's exclusive genetic architecture enables a coherent adaptive chain: multimetal resistance genes (*copF*, *copB*, *czcA*, nodT‐family efflux, molybdate transport) confer survival under Se, Cu, and Ni stress; QS‐independent biofilm regulation ensures EPS production is maintained under metal stress conditions that suppress QS‐dependent biofilm formation in other strains; and the resulting intact EPS matrix provides the structural scaffold for templated biosynthesis of crystalline t‐Se nanorods, a biotransformation product with properties superior to the amorphous nanospheres produced by all previously reported bacterial systems. This genotype–phenotype–nanomaterial linkage provides both a mechanistic framework and a rational foundation for bioprocess engineering of structure‐controlled Se nanomaterial production.

### 3.6 Study Limitations and Future Directions

Several limitations frame the interpretation of these findings and define the priorities for future work. First, the proposed link between individual exclusive genes (*copF*, *copB*, *czcA*, the Wzy‐family ligase, and the GH92 α‐mannosidase) and the nanorod‐templating phenotype rests on comparative genomics, structural modelling, and expression data; it is correlative, and direct causal attribution will require targeted gene knockout, complementation, and heterologous‐expression experiments, which constitute the immediate next step of this work. Second, although the three‐state experiment provides direct phenotypic evidence that an intact EPS matrix is required for efficient crystalline nanorod formation, the EPS itself has not yet been characterized biochemically; compositional glycomics and proteomics, together with direct structural analysis of the matrix–nanorod interface, are needed to test the templating model directly. Third, the allotropic outcome has been assessed qualitatively rather than quantitatively: nanorod yield, size and aspect‐ratio distributions, and crystalline phase fractions were not measured, and their systematic quantification will be essential to benchmark synthesis efficiency. Fourth, the metal‐tolerance and RT‐qPCR assays were performed under planktonic conditions as a first‐order validation of genomic content and do not directly report the EPS‐associated physiology that is the focus of this study. Finally, the biotechnological potential discussed here has not been evaluated at pilot scale: long‐term biofilm stability, process optimization and production yield, performance in complex real effluents, scalability, and the regulatory and techno‐economic aspects of deployment all remain to be assessed. BII‐R7 is therefore presented as a promising, mechanistically rationalized candidate accompanied by a defined experimental roadmap—spanning targeted mutagenesis, EPS characterization, quantitative crystallography, and immobilized‐cell bioreactor trials with techno‐economic analysis—rather than as a finished bioprocess.

## 4 Conclusions

*S. bentonitica* BII‐R7 emerges as a promising candidate for eco‐friendly applications in metal bioremediation and controlled nanomaterial production. This study addresses three previously unresolved questions in the biology of biogenic selenium nanomaterial formation. First, it provides direct phenotypic evidence that crystalline trigonal Se nanorod biosynthesis is not an intrinsic enzymatic property of *S. bentonitica* BII‐R7 but an emergent property of its biofilm EPS matrix: the same organism, without its EPS, produces mainly amorphous nanospheres. Second, it provides a genomic explanation for BII‐R7's capacity to maintain this matrix under polymetallic stress: an exclusive accessory genome of over 2300 genes, validated at the transcript level by RT‐qPCR and at the phenotypic level by Cu and Ni tolerance assays, encodes complementary metal resistance mechanisms (*copF*, *copB*, *czcA*, NodT‐family efflux, and molybdate transport) and replaces the AHL‐sensitive QS‐dependent biofilm circuit with a metal‐insensitive regulatory architecture. Third, two exclusive EPS‐remodelling genes, the Wzy‐family O‐antigen ligase GGMGOOPI_00064 and the secreted GH92 α‐mannosidase GGMGOOPI_00067, transcriptionally induced under Se stress and divergent from all homologues specifically in their substrate‐binding domains, are identified as the strongest genomic candidates for the structural determinants of the nanorod‐templating EPS architecture, providing specific and testable targets for future mutagenesis. Taken together, these findings provide a rational, genomics‐informed foundation for the future development of scalable crystalline Se nanomaterial production: preserving the biofilm is the key; the exclusive genetic toolkit of BII‐R7 is what makes preservation possible under the polymetallic conditions of real contaminated environments. The non‐pathogenic status of this organism (Risk Group 1) removes the primary deployment barrier, and its demonstrated compatibility with alginate hydrogel immobilization (González‐Morales et al. 2025) provides an immediately scalable delivery format for both bioremediation and green nanofabrication applications.

## Author Contributions

**Eduardo Perez‐Muelas:** writing – original draft, writing – review and editing, visualization, validation, methodology, investigation, formal analysis, conceptualization. **Miguel Angel Ruiz‐Fresneda:** writing – original draft, writing – review and editing, visualization, validation, supervision, methodology, formal analysis, conceptualization. **Guillermo Lazuen‐Lopez:** writing – review and editing, investigation, formal analysis. **Tatiana Lopez‐Perez:** investigation, formal analysis. **Fouad Eddaoudi‐Lakraichi:** investigation, formal analysis. **Mohammed Bakkali:** writing – review and editing, supervision, resources, formal analysis, conceptualization. **Mohamed Larbi Merroun:** writing – review and editing, resources, supervision, project administration, methodology, formal analysis, funding acquisition, conceptualization.

## Funding

This work was supported by grant B‐CIC‐006‐UGR23 funded by Consejería de Universidad, Investigación e Innovación and by ERDF Andalusia Program 2021‐2027, and TED2021‐131099B‐I00 funded by MICIU/AEI/10.13039/501100011033, Spain, and by the European Union's Horizon Europe WIDERA 2021 programme under the SURRI project (Grant Agreement No. 101079345).

## Conflicts of Interest

The authors declare no conflicts of interest.

## Supporting information


**Figure S1:** Visual comparison of selenium nanoparticle synthesis by *Stenotrophomonas bentonitica* BII‐R7 using three cellular modes: biofilms (left), planktonic cells (middle), and EPS‐depleted cells (right). The metallic grey coloration of the biofilm‐derived precipitate (left) is consistent with trigonal crystalline Se(0), in contrast to the bright red amorphous Se(0) observed in planktonic (middle) and EPS‐depleted (right) conditions, demonstrating the role of the intact EPS matrix in directing the allotropic outcome of Se(IV) biotransformation.
**Figure S2:** HAADF‐STEM micrographs and corresponding EDX spectra of biogenic selenium nanoparticles (BioSeNPs) produced by *S. bentonitica* in three physiological states: biofilm‐associated (A–B), planktonic (C–D), and EPS‐free cells (E–F).
**Figure S3:** Genes associated with biofilm formation in the four *Stenotrophomonas* strains. The isolates are shown on the left, and the biofilm‐forming genes are shown at the top. Dark blue colour indicates the presence of the gene, and light blue indicates its absence.
**Figure S4:** Basal biofilm formation in *Stenotrophomonas* strains. Quantification of biofilm production was performed by measuring absorbance at 595 nm in *S. chelatiphaga* DSM 21508, *S. pavanii* DSM 25135, *S. rhizophila* DSM 14405, and *S. bentonitica* BII‐R7.
**Table S1:** Genomic features of the *Stenotrophomonas* strains analysed in this study.
**Table S2:** Comparison of previous and newly generated genome assemblies for *Stenotrophomonas bentonitica* BII‐R7.
**Figure S5:** Heatmap of the Average Nucleotide Identity (ANI) analysis showing the percentage of identity among the different *Stenotrophomonas* strains included in the study.
**Figure S6:** Pangenome composition of *Stenotrophomonas* genomes. (A) Distribution of genes into core (670 genes), soft‐core (73 genes), shell (3766 genes), and cloud (21,559 genes) genome categories. (B) Accumulation curves for core and pangenome across the analysed genomes.
**Table S3:** Genomic island features and horizontal gene transfer (HGT) events in *Stenotrophomonas* strains.
**Figure S7:** Visualization of the genomic features of 38 *Stenotrophomonas* strains. The phylogenetic tree is based on the core genome SNPs alignment. The tracks from left to right show the strains, isolation source, antibiotic resistance and rpfF type. The pangenome matrix shows the presence of genes (in blue) in each genome.
**Table S4:** Exclusive genes identified in *Stenotrophomonas bentonitica* BII‐R7 and their associated resistance functions. Gene descriptions and pollutant classes were retrieved from the BacMet database.
**Figure S8:** Transcriptomic expression heatmap (TPM values) of key genes involved in metal stress response and selenium biotransformation in *Stenotrophomonas bentonitica* BII‐R7 under control and Se(VI) exposure conditions. Gene expression levels are represented as raw z‐scores of TPM values across three biological replicates. Data extracted from Pinel‐Cabello et al. (2023).
**Table S5:** Primers used for RT‐qPCR analysis of metal resistance genes in *S. bentonitica* BII‐R7. Forward (F) and reverse (R) primer sequences are shown (5′–3′), together with assay efficiency and coefficient of determination (R^2^). All primer efficiencies fall within the accepted 90%–110% range for relative quantification by the 2^−ΔΔCT^ method.
**Figure S9:** Boxplot of Cq values reference and target genes from RT‐qPCR analysis (*n* = 4). For each gene, the box represents the interquartile range (IQR) with the horizontal line indicating the median. The upper and lower bounds of the box denote the 75th and 25th percentiles, respectively. Whiskers extend to the 5th and 95th percentiles.

## Data Availability

The data that support the findings of this study are available from the corresponding author upon reasonable request.
