# Supplementary material for: Genomic Insights Into the Multimetal Resilience and Biofilm‐Templated Nanorod Biosynthesis of Stenotrophomonas bentonitica BII‐R7: Bioremediation and Green Nanotechnology Implications
Source: Microb Biotechnol. 2026 Sep 6;19(9):e70422. doi: 10.1111/1751-7915.70422 (PMC13547831; doi:10.1111/1751-7915.70422)
Supplement: Supplementary file 1 [file MBT2-19-e70422-s001.docx]

**Genomic Insights into the Multimetal Resilience and Biofilm-Templated Nanorod Biosynthesis of *Stenotrophomonas bentonitica* BII-R7: Bioremediation and Green Nanotechnology Implications**

Eduardo Pérez-Muelas^1,#^, Miguel Angel Ruiz-Fresneda^1^, Guillermo Lazuen-López^1^, Tatiana López Pérez^2^, Fouad Eddaoudi-Lakraichi^1^, Mohammed Bakkali^2^, Mohamed Larbi Merroun^1^

^1^Department of Microbiology, Campus Fuentenueva, University of Granada, 18071 Granada, Spain

^2^Department of Genetics,  Campus Fuentenueva, University of Granada, 18071 Granada, Spain

**^#^Corresponding author:** Eduardo Pérez-Muelas ([edupermue@ugr.es](mailto:edupermue@ugr.es))

**Supplementary Figure 1**

**Supplementary Figure 1.** **Visual comparison of selenium nanoparticle synthesis by *Stenotrophomonas bentonitica* BII-R7 using three cellular modes**: biofilms (left), planktonic cells (middle), and EPS-depleted cells (right). The metallic grey coloration of the biofilm-derived precipitate (left) is consistent with trigonal crystalline Se(0), in contrast to the bright red amorphous Se(0) observed in planktonic (middle) and EPS-depleted (right) conditions, demonstrating the role of the intact EPS matrix in directing the allotropic outcome of Se(IV) biotransformation.

**Supplementary Figure 2**

**Figure 2: HAADF-STEM micrographs and corresponding EDX spectra of biogenic selenium nanoparticles (BioSeNPs) produced by *S. bentonitica* in three physiological states:** biofilm-associated (A–B), planktonic (C–D), and EPS-free cells (E–F)

**Supplementary Figure 3**

**Supplementary Figure 3: Genes associated with biofilm formation in the four *Stenotrophomonas* strains**. The isolates are shown on the left, and the biofilm-forming genes are shown at the top. Dark blue color indicates the presence of the gene, and light blue indicates its absence.

**Supplementary Figure 4**

**Supplementary Figure 4. Basal biofilm formation in *Stenotrophomonas* strains.** Quantification of biofilm production was performed by measuring absorbance at 595 nm in *S. chelatiphaga* DSM 21508, *S. pavanii* DSM 25135, *S. rhizophila* DSM 14405, and *S. bentonitica* BII-R7.

**Supplementary Table 1**

**Table S1. Genomic features of the *Stenotrophomonas* strains analyzed in this study.**

The table presents key genomic metrics, including organism name, source of isolation, assembly accession number, number of protein-coding genes, contig N50 value, GC content (%), total number of contigs, total genome length, as well as estimated genome completeness and contamination levels.

| **Organism** | **Isolation**  **source** | **Assembly**  **accession** | **Protein-**  **coding**  **genes** | **Contig**  **N50 (bp)** | **GC**  **(%)** | **No.**  **contigs** | **Total**  **length (bp)** | **Complete-**  **ness (%)** | **Contam-**  **ination (%)** |
| --- | --- | --- | --- | --- | --- | --- | --- | --- | --- |
| ***Stenotrophomonas bentonitica* BII-R7** | **Soil** | **GCA_001753645.1** | **4,028** | **327,036** | **66.5** | **29** | **4,398,470** | **99.12** | **1.16** |
| *Stenotrophomonas indicatrix* JUb19 | Plant | GCA_013450245.1 | 4,054 | 4,577,105 | 66.5 | 2 | 4,592,581 | 99.68 | 1.02 |
| *Stenotrophomonas indicatrix* PierC1 | Soil | GCA_001499755.1 | 4,387 | 207,738 | 66.5 | 59 | 4,638,575 | 99.46 | 0.39 |
| *Stenotrophomonas maltophilia* 305SMAL | Human | GCA_001072195.1 | 4,321 | 204,025 | 66.5 | 40 | 4,732,922 | 99.27 | 0.24 |
| *Stenotrophomonas maltophilia* 4521 | Human | GCA_022751045.1 | 4,006 | 118,130 | 66.5 | 93 | 4,480,464 | 99.47 | 0.68 |
| *Stenotrophomonas maltophilia* AU12-09 | Human | GCA_000346445.1 | 4,196 | 69,081 | 66.5 | 125 | 4,547,300 | 99.03 | 0.25 |
| *Stenotrophomonas maltophilia* BurE1 | Soil | GCA_001499695.1 | 4,210 | 211,532 | 66.5 | 48 | 4,504,590 | 97.65 | 0.39 |
| *Stenotrophomonas maltophilia* C357 | Human | GCA_002799095.1 | 4,310 | 765,983 | 66.0 | 14 | 4,804,814 | 98.51 | 0.97 |
| *Stenotrophomonas maltophilia* CBF10-1 | Soil | GCA_001595975.1 | 3,982 | 231,617 | 66.5 | 121 | 4,556,616 | 99.12 | 0.62 |
| *Stenotrophomonas maltophilia* CF13 | Human | GCA_012971765.1 | 4,086 | 4,591,696 | 66.5 | 1 | 4,591,696 | 99.00 | 0.33 |
| *Stenotrophomonas maltophilia* E861 | Human | GCA_002799135.1 | 4,191 | 289,991 | 66.5 | 28 | 4,652,436 | 99.46 | 0.45 |
| *Stenotrophomonas maltophilia* E999 | Human | GCA_002799215.1 | 3,879 | 1,112,883 | 66.5 | 8 | 4,408,302 | 98.28 | 0.29 |
| *Stenotrophomonas maltophilia* EP13 | Plant | GCA_002799055.1 | 4,281 | 360,300 | 66.5 | 24 | 4,749,990 | 99.25 | 0.53 |
| *Stenotrophomonas maltophilia* EPM1 | Human | GCA_000344215.1 | 4,321 | 644,579 | 66.5 | 19 | 4,787,769 | 98.37 | 0.37 |
| *Stenotrophomonas maltophilia* FDAARGOS_325 | Human | GCA_002208885.2 | 4,369 | 4,851,512 | 66.5 | 1 | 4,851,512 | 98.91 | 0.78 |
| *Stenotrophomonas maltophilia* FDAARGOS_507 | Human | GCA_003812985.1 | 4,139 | 4,577,568 | 66.5 | 1 | 4,577,568 | 98.83 | 0.63 |
| *Stenotrophomonas maltophilia* FDAARGOS_649 | Human | GCA_008693985.1 | 4,130 | 4,625,884 | 66.5 | 1 | 4,625,884 | 98.51 | 0.30 |
| *Stenotrophomonas maltophilia* FDAARGOS_92 | Human | GCA_002951115.1 | 4,365 | 4,820,221 | 66.5 | 1 | 4,820,221 | 98.61 | 0.45 |
| *Stenotrophomonas maltophilia* G51 | Human | GCA_002799165.1 | 4,368 | 2,480,350 | 66.0 | 5 | 4,846,973 | 99.14 | 0.52 |
| *Stenotrophomonas maltophilia* ISMMS3 | Human | GCA_001274595.1 | 4,129 | 4,804,002 | 66.5 | 1 | 4,804,002 | 97.66 | 1.89 |
| *Stenotrophomonas maltophilia* JV3 | Plant | GCA_000223885.1 | 4,063 | 4,544,477 | 67.0 | 1 | 4,544,477 | 98.23 | 0.72 |
| *Stenotrophomonas maltophilia* K279a | Human | GCA_000072485.1 | 4,386 | 4,851,126 | 66.5 | 1 | 4,851,126 | 99.25 | 0.17 |
| *Stenotrophomonas maltophilia* LMG978 | Plant | GCA_001431665.1 | 3,907 | 83,392 | 67.0 | 109 | 4,484,616 | 97.85 | 1.28 |
| *Stenotrophomonas maltophilia* NCTC10257 | Human | GCA_900186865.1 | 4,555 | 5,004,261 | 66.0 | 1 | 5,004,261 | 98.37 | 0.83 |
| *Stenotrophomonas maltophilia* NCTC10258 | Human | GCA_900475405.1 | 3,988 | 4,481,118 | 66.5 | 1 | 4,481,118 | 99.12 | 0.15 |
| *Stenotrophomonas maltophilia* NCTC10498 | Human | GCA_900475685.1 | 4,184 | 4,661,352 | 66.5 | 1 | 4,661,352 | 99.46 | 0.39 |
| *Stenotrophomonas maltophilia* OUC_Est10 | Soil | GCA_002138415.1 | 4,125 | 4,668,743 | 66.5 | 1 | 4,668,743 | 98.86 | 0.87 |
| *Stenotrophomonas maltophilia* R551-3 | Plant | GCA_000020665.1 | 4,039 | 4,573,969 | 66.5 | 1 | 4,573,969 | 99.02 | 0.12 |
| *Stenotrophomonas maltophilia* SeITE02 | Plant | GCA_000613205.1 | 4,040 | 133,603 | 66.5 | 63 | 4,557,111 | 99.41 | 0.53 |
| *Stenotrophomonas maltophilia* SVIA2 | Soil | GCA_004346945.1 | 4,011 | 179,965 | 66.5 | 38 | 4,499,711 | 99.07 | 0.19 |
| *Stenotrophomonas maltophilia* TG_2006_STM1 | Human | GCA_002739005.1 | 3,813 | 111,485 | 67.0 | 64 | 4,348,898 | 98.20 | 0.25 |
| *Stenotrophomonas maltophilia* W18 | Soil | GCA_003030985.1 | 4,270 | 4,738,432 | 66.0 | 1 | 4,738,432 | 98.21 | 0.76 |
| *Stenotrophomonas pavanii* DSM_25135 | Plant | GCA_001431565.1 | 3,835 | 79,654 | 67.5 | 129 | 4,313,798 | 98.07 | 0.40 |
| *Stenotrophomonas rhizophila* CFBP13529 | Plant | GCA_005233605.1 | 3,950 | 1,148,755 | 67.5 | 12 | 4,626,295 | 98.91 | 2.15 |
| *Stenotrophomonas rhizophila* DSM14405 | Plant | GCA_000661955.1 | 3,938 | 736,911 | 67.5 | 8 | 4,648,976 | 98.68 | 2.48 |
| *Stenotrophomonas sp.* B1-1 | Soil | GCA_010894375.1 | 4,244 | 260,523 | 67.0 | 66 | 4,753,339 | 98.76 | 2.41 |
| *Stenotrophomonas sp.* LM091 | Plant | GCA_001806305.1 | 3,683 | 4,317,450 | 66.5 | 1 | 4,317,450 | 97.55 | 0.55 |
| *Stenotrophomonas sp.* OVS01A | Plant | GCA_019969775.1 | 4,072 | 884,132 | 66.5 | 25 | 4,553,025 | 99.74 | 0.76 |

bp, base pairs; GC, guanine–cytosine content.

**Supplementary Table 2**

**Table S2. Comparison of previous and newly generated genome assemblies for *Stenotrophomonas bentonitica* BII-R7.**

| **Assembly Quality Report** | **Previous Assembly [GCA_001753645_1_ASM175364v1]** | **New Assembly**  **[PRJNA1232115]** |
| --- | --- | --- |
| **# Contigs (>= 1000 bp)** | 191 | 22 |
| **Total length (>= 1000 bp)** | 4,371,992 | 4,395,616 |
| **Largest contig** | 187,875 | 722,064 |
| **GC (%)** | 66.46 | 66.47 |
| **N50** | 35,432 | 327,036 |
| **N75** | 22,070 | 205,857 |
| **L50** | 38 | 4 |
| **L75** | 76 | 8 |
| **N's per 100 kbp** | 0.00 | 0.00 |
| **CheckM Completeness** | 94.28% | 99.12% |
| **CheckM Contamination** | 2.28% | 1.16% |

**Supplementary Figure 5**

**Supplementary Figure 5.** Heatmap of the Average Nucleotide Identity (ANI) analysis showing the percentage of identity among the different *Stenotrophomonas* strains included in the study.

**Supplementary Figure 6**

**Supplementary Figure 6. Pangenome composition of *Stenotrophomonas* genomes.** (A) Distribution of genes into core (670 genes), soft-core (73 genes), shell (3,766 genes), and cloud (21,559 genes) genome categories. (B) Accumulation curves for core and pangenome across the analyzed genomes.

**Supplementary Table 3**

**Table S3. Genomic island features and horizontal gene transfer (HGT) events in *Stenotrophomonas* strains.**

This table summarizes key genomic features for each *Stenotrophomonas* strain, including the total number of genomic islands, HGT events, total island size, average island size, and source of isolation for each strain (human, plant, or soil).

| **Organism** | **No.**  **islands** | **Islands with**  **homology** | **Islands with HGT**  **signature + homology** | **Total island**  **size (bp)** | **Avg. island**  **size (bp)** | **Genome**  **length (bp)** | **Isolation**  **source** |
| --- | --- | --- | --- | --- | --- | --- | --- |
| *Stenotrophomonas maltophilia* 305SMAL | 40 | 6 | 3 | 288,000 | 7,200 | 4,732,922 | Human |
| *Stenotrophomonas maltophilia* 4521 | 42 | 1 | 0 | 208,000 | 4,952 | 4,480,464 | Human |
| *Stenotrophomonas maltophilia* AU12-09 | 44 | 27 | 18 | 280,000 | 6,363 | 4,547,300 | Human |
| *Stenotrophomonas maltophilia* C357 | 45 | 12 | 11 | 264,000 | 5,866 | 4,804,814 | Human |
| *Stenotrophomonas maltophilia* CF13 | 36 | 0 | 0 | 246,000 | 6,833 | 4,591,696 | Human |
| *Stenotrophomonas maltophilia* E861 | 25 | 1 | 0 | 158,000 | 6,320 | 4,652,436 | Human |
| *Stenotrophomonas maltophilia* E999 | 38 | 13 | 7 | 214,000 | 5,631 | 4,408,302 | Human |
| *Stenotrophomonas maltophilia* EPM1 | 40 | 3 | 1 | 292,000 | 7,300 | 4,787,769 | Human |
| *Stenotrophomonas maltophilia* FDAARGOS_325 | 42 | 0 | 0 | 292,000 | 6,952 | 4,851,512 | Human |
| *Stenotrophomonas maltophilia* FDAARGOS_507 | 40 | 0 | 0 | 246,000 | 6,150 | 4,577,568 | Human |
| *Stenotrophomonas maltophilia* FDAARGOS_649 | 38 | 0 | 0 | 210,000 | 5,526 | 4,625,884 | Human |
| *Stenotrophomonas maltophilia* FDAARGOS_92 | 44 | 0 | 0 | 260,000 | 5,909 | 4,820,221 | Human |
| *Stenotrophomonas maltophilia* G51 | 38 | 10 | 7 | 346,000 | 9,105 | 4,846,973 | Human |
| *Stenotrophomonas maltophilia* ISMMS3 | 41 | 0 | 0 | 260,000 | 6,341 | 4,804,002 | Human |
| *Stenotrophomonas maltophilia* K279a | 42 | 0 | 0 | 244,000 | 5,809 | 4,851,126 | Human |
| *Stenotrophomonas maltophilia* NCTC10257 | 46 | 1 | 1 | 290,000 | 6,304 | 5,004,261 | Human |
| *Stenotrophomonas maltophilia* NCTC10258 | 35 | 0 | 0 | 172,000 | 4,914 | 4,481,118 | Human |
| *Stenotrophomonas maltophilia* NCTC10498 | 43 | 0 | 0 | 236,000 | 5,488 | 4,661,352 | Human |
| *Stenotrophomonas maltophilia* TG_2006_STM1 | 42 | 5 | 2 | 206,000 | 4,904 | 4,348,898 | Human |
| *Stenotrophomonas indicatrix* JUb19 | 43 | 25 | 17 | 214,000 | 4,976 | 4,592,581 | Plant |
| *Stenotrophomonas maltophilia* EP13 | 40 | 0 | 0 | 260,000 | 6,500 | 4,749,990 | Plant |
| *Stenotrophomonas maltophilia* JV3 | 45 | 0 | 0 | 206,000 | 4,577 | 4,544,477 | Plant |
| *Stenotrophomonas maltophilia* LMG978 | 41 | 8 | 1 | 254,000 | 6,195 | 4,484,616 | Plant |
| *Stenotrophomonas maltophilia* R551-3 | 37 | 0 | 0 | 182,000 | 4,918 | 4,573,969 | Plant |
| *Stenotrophomonas maltophilia* SeITE02 | 45 | 31 | 23 | 212,000 | 4,711 | 4,557,111 | Plant |
| *Stenotrophomonas pavanii* DSM_25135 | 38 | 26 | 1 | 180,000 | 4,736 | 4,313,798 | Plant |
| *Stenotrophomonas rhizophila* CFBP13529 | 42 | 18 | 1 | 220,000 | 5,238 | 4,626,295 | Plant |
| *Stenotrophomonas rhizophila* DSM14405 | 49 | 0 | 0 | 198,000 | 4,040 | 4,648,976 | Plant |
| *Stenotrophomonas sp.* LM091 | 22 | Not classified | Not classified | 238,000 | 10,818 | 4,317,450 | Plant |
| *Stenotrophomonas sp.* OVS01A | 46 | Not classified | Not classified | 218,000 | 4,739 | 4,553,025 | Plant |
| ***Stenotrophomonas bentonitica* BII-R7** | **35** | **34** | **8** | **222,000** | **6,342** | **4,398,470** | **Soil** |
| *Stenotrophomonas indicatrix* PierC1 | 55 | 29 | 13 | 262,000 | 4,763 | 4,638,575 | Soil |
| *Stenotrophomonas maltophilia* BurE1 | 39 | 3 | 1 | 230,000 | 5,897 | 4,504,590 | Soil |
| *Stenotrophomonas maltophilia* CBF10-1 | 42 | 4 | 1 | 248,000 | 5,904 | 4,556,616 | Soil |
| *Stenotrophomonas maltophilia* OUC_Est10 | 46 | 1 | 1 | 244,000 | 5,304 | 4,668,743 | Soil |
| *Stenotrophomonas maltophilia* SVIA2 | 42 | 0 | 0 | 262,000 | 6,238 | 4,499,711 | Soil |
| *Stenotrophomonas maltophilia* W18 | 39 | 0 | 0 | 242,000 | 6,205 | 4,738,432 | Soil |
| *Stenotrophomonas sp.* B1-1 | 48 | Not classified | Not classified | 276,000 | 5,750 | 4,753,339 | Soil |

bp, base pairs; HGT, horizontal gene transfer; No., number. 'Not classified': strains lacking sufficient reference data for island classification.

**Supplementary Figure 7**

**Supplementary Figure 7 Visualization of the genomic features of 38 *Stenotrophomonas* strains**. The phylogenetic tree is based on the core genome SNPs alignment. The tracks from left to right show the strains, isolation source, antibiotic resistance and rpfF type. The pangenome matrix shows the presence of genes (in blue) in each genome.

**Supplementary Table 4**

**Table S4. Exclusive genes identified in *Stenotrophomonas bentonitica* BII-R7 and their associated resistance functions. Gene descriptions and pollutant classes were retrieved from the BacMet database.**

| **Query**  **accession** | **BacMet**  **description** | **Identity**  **(%)** | **E-value** | **Polluting agent (compound class)** |
| --- | --- | --- | --- | --- |
| **GGMGOOPI_00060** | Protein-L-isoaspartate O-methyltransferase | 100 | 0 | Sodium Dodecyl Sulfate (SDS) *[class: Organo-sulfate]*  Carbonyl cyanide 3-chlorophenylhydrazone (CCCP) *[class: Hydrazone]*  Acriflavine *[class: Acridine]* |
| **GGMGOOPI_00180** | Multidrug resistance outer membrane protein SmeF | 100 | 0 | Triclosan *[class: Phenolic compounds]*  Acriflavine *[class: Acridine]*  Rhodamine 6G *[class: Xanthene]*  Crystal Violet *[class: Triarylmethane]*  Ethidium Bromide *[class: Phenanthridine]*  Proflavin *[class: Acridine]*  SDS *[class: Organo-sulfate]* |
| **GGMGOOPI_00882** | Copper-translocating P-type ATPase (CopF) | 90.47 | 0 | Copper (Cu) *[class: Metal]* |
| **GGMGOOPI_01176** | Molybdate ABC transporter inner membrane subunit | 100 | 6.4 × 10⁻¹²⁰ | Tungsten (W) *[class: Metal]*  Molybdenum (Mo) *[class: Metal]* |
| **GGMGOOPI_01395** | Copper resistance protein B | 82.76 | 1.53 × 10⁻¹⁶⁵ | Copper (Cu) *[class: Metal]* |
| **GGMGOOPI_03943** | NodT family RND efflux system outer membrane lipoprotein | 100 | 0 | Carbonyl cyanide 3-chlorophenylhydrazone (CCCP) *[class: Hydrazone]*  Tetrachlorosalicylanilide (TCS) *[class: Salicylanilide]* |
| **GGMGOOPI_03944** | EmrRCABsm operon negative regulator EmrRsm (MarR-type) | 100 | 2.69 × 10⁻¹⁰⁰ | Carbonyl cyanide 3-chlorophenylhydrazone (CCCP) *[class: Hydrazone]*  Tetrachlorosalicylanilide (TCS) *[class: Salicylanilide]* |
| **GGMGOOPI_02614** | ABC-type multidrug efflux pump protein SmrA | 79.09 | 8.79 × 10⁻¹⁵⁸ | Ethidium Bromide *[class: Phenanthridine]*  Rhodamine 6G *[class: Xanthene]*  Tetraphenylphosphonium (TPP) *[class: Quaternary Ammonium Compounds (QACs)]*  4,6-diamidino-2-phenylindole (DAPI) *[class: Diamidine]* |
| **GGMGOOPI_01175** | Molybdenum ABC transporter ATPase subunit (ModABC complex) | 75.34 | 1.05 × 10⁻¹¹⁶ | Tungsten (W) *[class: Metal]*  Molybdenum (Mo) *[class: Metal]* |

**ABC, ATP-binding cassette; CCCP, carbonyl cyanide 3-chlorophenylhydrazone; DAPI, 4,6-diamidino-2-phenylindole; QACs, quaternary ammonium compounds; RND, resistance-nodulation-division; SDS, sodium dodecyl sulfate; TCS, tetrachlorosalicylanilide; TPP, tetraphenylphosphonium. E-values of 0 indicate values below ~10⁻³²⁰.**

**Supplementary Figure 8**

**Supplementary Figure 8 Transcriptomic expression heatmap (TPM values) of key genes involved in metal stress response and selenium biotransformation in *Stenotrophomonas bentonitica* BII-R7 under control and Se(VI) exposure conditions.** Gene expression levels are represented as raw z-scores of TPM values across three biological replicates. Data extracted from Pinel-Cabello et al., (2021)

**Supplementary Table 5**

**Table S5**. Primers used for RT-qPCR analysis of metal resistance genes in *S. bentonitica* BII-R7. Forward (F) and reverse (R) primer sequences are shown (5′–3′), together with assay efficiency and coefficient of determination (R²). All primer efficiencies fall within the accepted 90–110% range for relative quantification by the 2^(−ΔΔCT) method.

| **Target gene** | **Primer** | **Assay linearity** |
| --- | --- | --- |
| *czcA* | F: ATGGCGGTGGTGATCATGTT | Efficiency: 100.6%  R^2^ : 0,986 |
|  | R: AGACTTCTGCCTTGGTCTGC |  |
| *copF* | F: CGGTGATCATCTCGCTCACA | Efficiency: 109.3%  R^2^ : 0,993 |
|  | R: CGATCTCCACGTCTTCCTCG |  |
| *copB* | F: TTGATCAACCGCCTGGAACA | Efficiency: 97.5%  R^2^ : 0,997 |
|  | R: GCCCGTACAACACTTCCAGA |  |
| *16s* | F: AGAGTTGATCCTGGCTCAG | Efficiency: 93.7%  R^2^ : 0,98 |
|  | R: GGTTACCTTGTTACGACTT |  |

**Supplementary Figure 9**

**Supplementary Figure 9. Boxplot of Cq values reference and target genes from RT-qPCR analysis (n = 4)**. For each gene, the box represents the interquartile range (IQR) with the horizontal line indicating the median. The upper and lower bounds of the box denote the 75th and 25th percentiles, respectively. Whiskers extend to the 5th and 95th percentiles.
